# Clinical features and endoscopic management of sharp wooden object ingestions: a systematic review of 479 cases

**DOI:** 10.1093/gastro/goaf035

**Published:** 2025-05-14

**Authors:** Kay Chen, Benjamin Chipkin, Alyssa A Grimshaw, Fateh Bazerbachi, Darrick K Li

**Affiliations:** Section of Digestive Diseases, Department of Medicine, Yale School of Medicine, New Haven, CT, USA; Section of Digestive Diseases, Department of Medicine, Yale School of Medicine, New Haven, CT, USA; Harvey Cushing/John Hay Whitney Medical Library, Yale University, New Haven, CT, USA; Interventional Endoscopy Program, St Cloud Hospital, CentraCare Health System, St Cloud, MN, USA; Section of Digestive Diseases, Department of Medicine, Yale School of Medicine, New Haven, CT, USA

**Keywords:** toothpick, wooden objects, endoscopy, foreign body ingestion

## Abstract

**Background and aims:**

Ingested sharp objects pose increased risks of adverse events compared with other foreign bodies. We conducted the largest systematic review to date of sharp wooden object ingestions to elucidate patterns in clinical presentation and guide management practices.

**Methods:**

Cochrane Library, Google Scholar, Ovid MEDLINE, Ovid Embase, PubMed, Scopus, and Web of Science Core Collection databases were searched for cases of adults who ingested sharp wooden objects. Descriptive statistics were reported for risk factors, clinical presentations, laboratory and imaging findings, adverse events, and treatments.

**Results:**

Analysis of 479 cases showed that most patients were unaware of ingestion (74.8%) and toothpicks were the most common ingested item (92.5%). Male sex (70.1%), substance use (9.4%), and edentulousness (6.1%) were risk factors. Common symptoms included abdominal pain (83.7%) and fever (36.7%). Imaging identified the object in 48.1% of cases, with computed tomography being the most sensitive (54.7%). Objects were commonly found in the gastrointestinal tract (79.3%). They were consistently found in the gastrointestinal tract when patients were aware of ingestion or imaging showed an intraluminal/transluminal location. Endoscopy visualized the objects 76.1% of the time, with successful removal in 88.8% of cases; 4.7% of patients required surgery following endoscopic removal. Adverse events included perforation (87.5%) and abscess (33.0%), with a mortality rate of 5.0%.

**Conclusions:**

Ingestion of sharp wooden objects presents heterogeneously and can lead to serious complications. Endoscopic removal is safe and effective. We propose a clinical algorithm to guide physicians in diagnosing and managing suspected sharp wooden object ingestion.

## Introduction

Foreign body ingestions are a common reason for hospital presentation, accounting for >165,000 emergency room visits in the USA in 2018 [1]. While most foreign bodies pass spontaneously through the gastrointestinal (GI) tract without incident [2], sharp or pointed objects pose an increased risk of adverse events, including perforation, abscess, GI bleeding, or death [3, 4]. Pointed wooden objects such as toothpicks are ubiquitous and easily swallowed, yet the hazards posed by these items are frequently overlooked.

Management approaches for ingested sharp objects remain varied, with debate surrounding the optimal use of conservative observation, endoscopic retrieval, or surgical removal [4–8]. The lack of consensus stems from limited large-scale studies to guide best practices. This systematic review aims to elucidate the patterns of clinical presentation and outcomes associated with ingestion of sharp wooden objects. We chose to evaluate sharp wooden objects and use this as a surrogate for all sharp pointed objects because they are frequently used and easily swallowed, not well visualized on imaging, and unique enough to be reported in the literature. By analysing the largest cohort of cases to date, we seek to identify key factors that can inform management decisions and propose a clinical algorithm for approaching these challenging scenarios.

## Methods

This systematic review was conducted according to a published protocol on PROSPERO (CRD42022369469) and followed the Preferred Reporting Items for Systematic Reviews and Meta-Analysis and Synthesis Without Meta-analysis in Systematic Review reporting guidelines (Supplementary Tables S1–S3) [9, 10].

### Eligibility criteria

The inclusion criteria included patients who were aged ≥18 years and had ingested wooden toothpicks, lollipop sticks, chopsticks, popsicle sticks, dental picks, skewers, cocktail sticks, sandwich spears, or other sharp wooden objects.

### Search strategy and information sources

A medical librarian (A.A.G.) searched the following databases: Cochrane Library, Google Scholar, Ovid MEDLINE, Ovid Embase, PubMed, Scopus, and Web of Science Core Collection from the inception of each database to 5 January 2024 (Supplementary Table S4) by using controlled vocabulary and keywords for the foreign bodies of interest. The search was peer-reviewed by a second librarian using the Peer Review for Electronic Search Strategies [11]. CitationChaser was used to compile citations from reference lists and studies that cite the included studies to find additional relevant studies that were not retrieved by using the database searches [12].

### Study selection

Results from the search were imported into the Endnote 20 database. After duplicates were removed by using the Yale Reference Deduplicator, the remaining studies were uploaded into Covidence [13]. Study selection, data extraction, and risk-of-bias assessment were made by two independent reviewers (K.C. and B.C.) and disagreements were settled by discussion and adjudication by the corresponding author (D.K.L.). Case reports and case series in all publication formats (full texts, letters to the editor, abstracts, images, or videos) in all languages were included.

### Data collection and data items

Data used included the year of publication, publication form, publication language, country of origin, age, sex, race, ethnicity, medical history, symptoms at the time of presentation, laboratory findings, radiology reports, details of endoscopic and/or surgical treatment, object ingested, knowledge of ingestion and circumstances around ingestion, time from ingestion to diagnosis, adverse events from ingestion, post-treatment adverse events, use of antibiotics, location of object in the body, number of presentations before diagnosis, and final outcome. Patients were considered to have leukocytosis or an elevated C-reactive protein if the value was reported to be >11.0 × 10^3^/µL or >3 mg/L, respectively, or if they were reported qualitatively to be elevated. Anemia was defined as a hemoglobin of <12 g/dL or hematocrit of <35% in all cases for simplicity, as biologic sex was not reported in all cases. In addition, patients were considered to have anemia if this was reported qualitatively. To ensure consistency, data were initially extracted by reviewers from a pilot group of 10 studies with a concordance of 90%.

### Risk-of-bias assessment

The methodological quality and synthesis of case series and case reports tool was used to evaluate included reports (Supplementary Table S5) [14] and this tool has been applied previously with consistency among reviewers [15–17].

### Outcomes of interest

Our primary outcome of interest was the development of adverse events after ingestion of sharp wooden objects. Adverse events were defined as events related to injury of the gastrointestinal mucosa due to ingestion including but not limited to perforation, abscess, bacteremia, gastrointestinal bleeding, and obstruction. Secondary outcomes of interest included location of the ingested object, safety of endoscopy for removal, need for surgery, need for antibiotics, and mortality.

### Statistical analysis

For descriptive analysis, we reported medians and interquartile ranges for continuous variables and percentages for dichotomized variables unless otherwise specified. A comparison of outcomes was performed by using the Pearson’s χ^2^ test. When the number of patients in any cell in a contingency table was under five, we used the Fisher’s exact test. Two-tailed *P*-values were statistically significant when they were <0.05. Multivariate logistic regression was used to determine covariates associated with the development of adverse events in patients with sharp wooden object ingestion adjusting for age, sex, antibiotic use, performance of endoscopy, and awareness of ingestion. All statistical analyses were conducted by using R 4.4.0 (R Foundation for Statistical Computing, Vienna, Austria).

## Results

### Study characteristics

**Figure 1.**
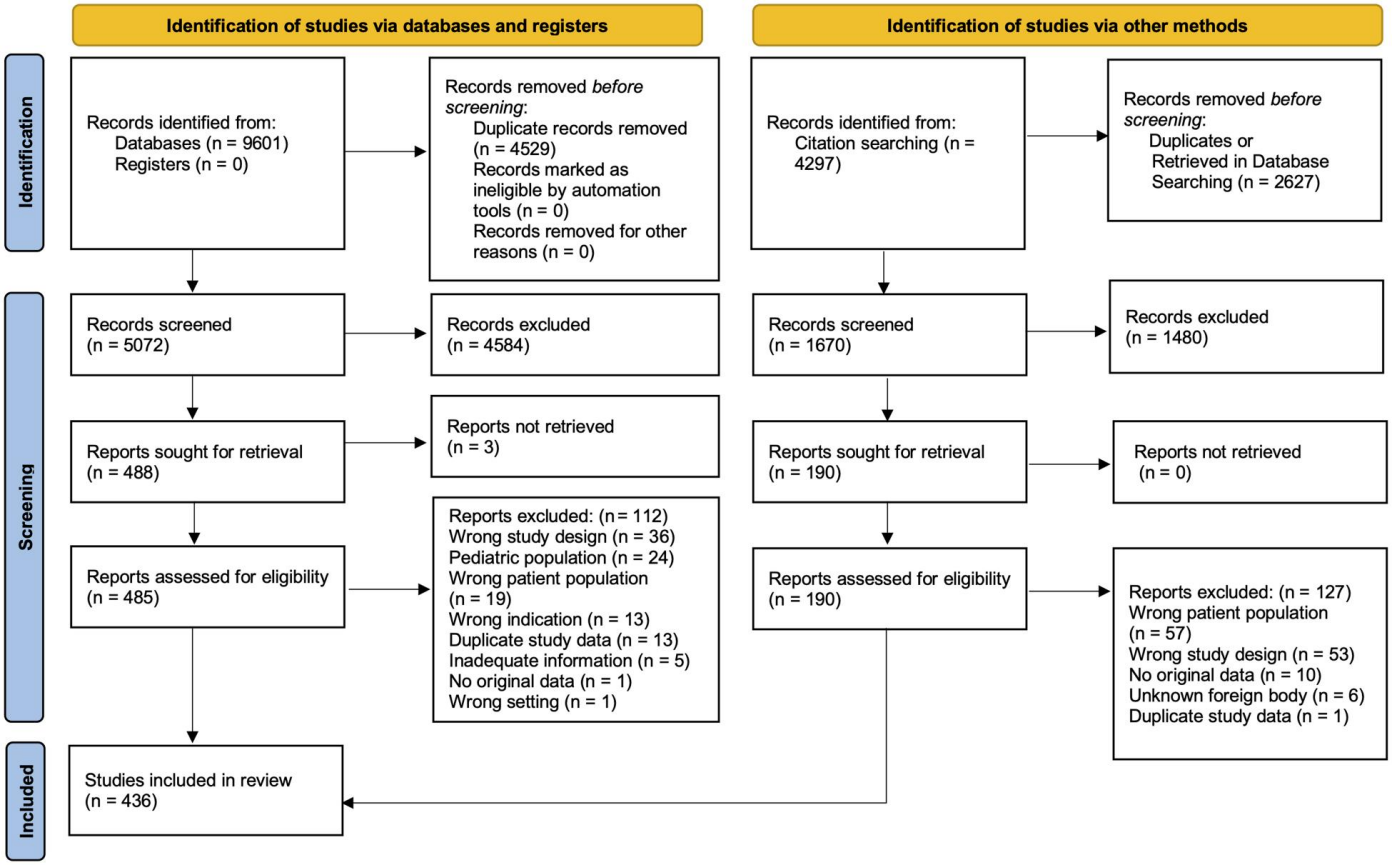
PRISMA flow chart for systematic review. Adapted from: Page MJ, McKenzie JE, Bossuyt PM, *et al*. The PRISMA 2020 statement: an updated guideline for reporting systematic reviews. *BMJ*. 2021;372:n71. doi: 10.1136/bmj.n71.

Database searches resulted in 9,601 citations (Figure 1). After duplicates were removed, 5,072 citations underwent title and abstract screening. Of these, 488 studies were sought for retrieval and 3 manuscripts were unable to be located (Supplementary Table S6). Four hundred and eighty-five studies were reviewed in full, with 112 being eliminated based on exclusion criteria of wrong study design, pediatric populations, wrong patient population, wrong indication, duplicate study data, inadequate information, and no original data (Supplementary Table S7). An additional 63 references were found through citations chasing. In total, 436 studies comprising 479 cases were included in the study. These studies were published in 12 different languages from 55 different countries/areas (Supplementary Table S8). The publication date ranged from 1910 to 2024 (Supplementary Table S9). Most studies were full texts (82.0%), with the remainder comprising abstracts, letters to the editor, or images (Supplementary Table S9).

### Assessment of methodological quality of included studies

Reviewer agreement in the methodological quality of the included studies was 97.0%. Most studies showed an unclear risk for selection bias and a low risk of ascertainment and causality bias (>95.0%); 44.4% of studies had a high risk of reporting bias and 55.6% of studies had unclear risk of reporting bias (Supplementary Figure S1).

### Patient characteristics

The demographics and details of patients with sharp wooden object ingestion are reported in Table 1. The median age was 54 years [range 18–93, interquartile range (42, 64)]. Most patients were male (70.1%). Race and ethnicity were only reported in 49 cases, but the majority of these were White/Caucasian (73.5%). Substance-use disorder (9.4%) and edentulousness (6.1%) were the most-reported comorbidities. The most common object ingested was a toothpick (92.5%) followed by skewers (2.3%) and chopsticks (2.1%).

**Table 1.** Patient demographics and details of sharp wooden object ingestions in 479 cases

Variables	*n* (%)
Age, years, median (IQR)^a^	54 (42, 64)
Male^b^	333 (70.1)
Race/ethnicity^c^	
White/Caucasian	36 (73.5)
Black	8 (16.3)
Asian	4 (8.2)
Hispanic	1 (2.0)
Comorbidities	
Substance-use disorder	46 (9.6)
Edentulous	29 (6.1)
Depression	8 (1.7)
Psychosis	5 (1.0)
Intellectual disability	4 (0.8)
Dementia	4 (0.8)
Pica	2 (0.4)
Anxiety	2 (0.4)
Sharp wooden object ingested	
Toothpick	443 (92.5)
Skewer	11 (2.3)
Chopstick	10 (2.1)
Cocktail stick	8 (1.7)
Wood stick needle	3 (0.6)
Lollipop stick	1 (0.2)
Wooden bowel fragment	1 (0.2)
Matchstick	1 (0.2)
Pointy wood shard	1 (0.2)
Knowledge of ingestion^d^	
Aware at time of presentation	44 (13.5)
Recalled ingestion after diagnosis	38 (11.7)
Unaware	243 (74.8)

IQR = interquartile range.

a The number of cases was 473.

b The total number of cases was 475, including 333 males and 142 females.

c The number of cases was 49.

d The number of cases was 325.

### Knowledge of ingestion

The presence or lack of knowledge regarding foreign body ingestion by the patient at the time of presentation was not specifically reported in 154 out of 479 cases (32.2%). Of the remaining 325 cases, lack of awareness was reported for 243 patients (74.8%) while 44 patients (13.5%) were aware of the ingestion at the time of initial presentation. Thirty-eight patients (11.7%) recalled the ingestion retrospectively after the diagnosis was made and 22.1% (104/471) of patients required multiple clinical encounters with medical providers before a diagnosis was made. Ingestion was often related to eating or drinking items that contained the foreign body (29.5%, 96/325) or drinking alcohol (10.5%, 34/325). Fifteen patients (4.6%) reported a habit of chewing on toothpicks and seven patients (2.2%) reported often falling asleep with toothpicks in their mouth. In total, 76.8% (63/82) of those who were aware of ingesting the object were aware of the precise time at which the ingestion occurred relative to their presentation. In this group, the median number of days after ingestion to diagnosis was 14 days but with a range from 12 hours to 61 years.

### Symptoms and laboratory findings

At the time of clinical presentation, most patients reported symptoms (99.2%), with the most frequent being abdominal pain (83.7%), vomiting (17.1%), and nausea (13.8%). In terms of presenting vital signs, 36.7% had fevers, 14.2% had tachycardia, and 9.4% had hypotension. Reported laboratory tests revealed leukocytosis in 67.1% of cases, anemia in 37.0% of cases, and elevated C-reactive protein in 91.0% of cases. These findings are summarized in Table 2.

**Table 2.** Symptoms, signs, and laboratory data of 479 patients with sharp wooden object ingestion

Variables	*n* (%)
Symptoms	
Any symptoms present	475 (99.2)
Abdominal pain	401 (83.7)
Vomiting	82 (17.1)
Nausea	66 (13.8)
Lack of appetite	36 (7.5)
Weight loss	28 (5.8)
Chills	25 (5.2)
Diarrhea	24 (5.0)
Lower urinary tract symptoms	15 (3.1)
Back pain	15 (3.1)
Constipation	14 (2.9)
Shortness of breath	13 (2.7)
Rectal pain	13 (2.7)
Fatigue/weakness	13 (2.7)
Cough	11 (2.3)
Altered mental status	11 (2.3)
Dysphagia	7 (1.5)
Hemoptysis	3 (0.6)
Heartburn	2 (0.4)
Signs	
Fever	176 (36.7)
Tachycardia	68 (14.2)
Hypotension	45 (9.4)
Laboratory data	
Leukocytosis^a^	190 (67.1)
Anemia^b^	47 (37.0)
Elevated C-reactive protein^c^	111 (91.0)

a The number of cases was 283.

b The number of cases was 127.

c The number of cases was 122.

### Location of ingested sharp objects

The reported locations in which the ingested objects were found are described in Table 3. The most common location was the GI tract (380 cases, 79.3%), with the stomach (19.2%), duodenum (14.4%), and colon (25.1%) being the most frequently involved locations within the GI tract. Of note, in the three cases in which the object was identified in the GI tract and diagnosis was made within 1 day of ingestion, the object was in the stomach. Beyond this time frame, the object could be found in any part of the GI tract. The liver was involved in 57 cases (11.9%). The most common other locations in which the sharp wooden object was found are outlined in Table 3, with rarer locations summarized in Supplementary Table S10. Notable locations include vascular involvement with the iliac arteries (2.7%) and inferior vena cava (1.7%) affected, as well as cardiac involvement (1.7%). In 122 patients, the object involved multiple organs often because of perforation into adjacent structures.

**Table 3.** Summary of the most common determined locations in which the ingested foreign body was found

Location	*n* (%)
Gastrointestinal tract	380 (79.3)
Esophagus	6 (1.3)
Stomach	92 (19.2)
Small bowel	142 (29.6)
Duodenum	69 (14.4)
Jejunum	22 (4.6)
Ileum	41 (8.6)
Unspecified small bowel location	10 (2.1)
Ileocecal valve	21 (4.4)
Appendix	5 (1.0)
Colorectal	131 (27.3)
Colon	120 (25.1)
Rectum	11 (2.3)
Liver	57 (11.9)
Others	161 (33.6)
Peritoneal cavity	17 (3.5)
Iliac artery^a^	13 (2.7)
Abdominal wall	13 (2.7)
Pancreas	10 (2.1)
Inferior vena cava	8 (1.7)
Heart^b^	8 (1.7)
Retroperitoneal space	8 (1.7)
Psoas muscle^c^	8 (1.7)
Right kidney	7 (1.5)
Mesentery	7 (1.5)
Omentum	6 (1.3)
Sacrum	5 (1.0)
Biliary tract^d^	5 (1.0)
More than one location	122 (25.5)

a Right external, *n *=* *3; right internal, *n *=* *2; right common, *n *=* *4; left external, *n *=* *1; left internal, *n *=* *1; left common, *n *=* *1; common of unknown laterality, *n *=* *1.

b Pericardium, *n *=* *4; right atrium, *n *=* *4; right ventricle, *n *=* *1; ventricle of unknown laterality, *n *=* *1.

c Right side, *n *=* *6; left side, *n *=* *2.

d Gallbladder, *n *=* *4; common bile duct, *n *=* *1.

### Adverse events

The most-reported adverse events related to ingestion were perforation (419/479, 87.5%) and abscess (158/479, 33.0%) (Table 4). Other notable adverse events that were noted included bacteremia (40/479, 8.4%), GI bleeding (48/479, 10.0%), inflammatory mass/pseudotumor (23/479, 4.8%), and fistula (21/479, 4.4%). Compared with patients who did not develop adverse events, patients who developed adverse events were older (53.3 vs 48.2 years, *P *=* *0.10) and more likely to be male (71.3% vs 51.7%, *P *=* *0.03). A summary of baseline characteristics in patients who did and did not develop adverse events as a result of their foreign body ingestion are provided in Supplementary Table S11. In adjusted logistic regression analysis, patients who were aware of ingestion were less likely to develop complications [odds ratio (OR) 0.31, 95% confidence interval (CI) 0.11–0.93, *P *=* *0.03]. In addition, patients who underwent endoscopy were also less likely to develop adverse events (OR 0.07, 95% CI 0.01–0.25, *P *<* *0.01). Male sex was associated with a non-significant trend toward increased risk of adverse events (OR 0.43, 95% CI 0.16–1.13, *P *=* *0.09).

**Table 4.** Overview of the common adverse events seen as a result of the ingested foreign body

Variable	*n* (%)
No adverse events	31 (6.5)
Gastrointestinal bleeding	48 (10.0)
Hematochezia	27 (5.6)
Melena	14 (2.9)
Hematemesis	12 (2.5)
Coffee ground emesis	1 (0.2)
Undefined gastrointestinal hemorrhage	1 (0.2)
Abscess	158 (33.0)
Bacteremia	40 (8.4)
Perforation	419 (87.5)
Others	154 (32.2)
Mass/pseudotumor	23 (4.8)
Fistula	21 (4.4)
Bowel obstruction	15 (3.1)
Appendicitis	10 (2.1)
Cardiac issue^a^	10 (2.1)
Septic thrombophlebitis	9 (1.9)
Soft skin or tissue infection^b^	9 (1.9)
Pulmonary issue^c^	8 (1.7)
Urinary tract infection	6 (1.3)
Pancreatitis	5 (1.0)
More than one adverse event	274 (57.2)

a Myocardial infarction, *n *=* *2; cardiac tamponade, *n *=* *2; pericarditis, *n *=* *2; endocarditis, *n *=* *3; atrial fibrillation, *n *=* *1; right atrial thrombus, *n *=* *1; and right coronary artery perforation, *n *=* *1.

b Cellulitis, *n *=* *4; Fournier’s gangrene, *n *=* *1; necrotizing fasciitis, *n *=* *4.

c Septic pneumonia, *n *=* *2; right-sided empyema, *n *=* *1; pleural effusion, *n *=* *1; pulmonary emboli (both septic, *n *=* *3; bland, *n *=* *1); and hemoptysis, *n *=* *1.

### Imaging findings

In total, 81.6% (391/479) of cases utilized imaging for diagnostic purposes. The foreign body was identified on imaging in 48.1% of these cases (Table 5). The most common imaging modality used was computed tomography (CT), with 285 cases performing at least one CT prior to foreign body removal. The foreign body was identified on CT in 54.7% of these cases. Ultrasounds were performed at least once in 32.8% of cases prior to foreign body removal and diagnostic in 28.7% of these cases. Magnetic resonance imaging was uncommonly ordered (5.2%) and was only diagnostic 16.0% of the time. Finally, although X-rays were ordered in 37.2% of cases, they were only diagnostic for foreign body ingestion 7.3% of the time and most of the positive studies involved contrast.

**Table 5.** Summary of the diagnostic imaging tests that were performed and their diagnostic ability

Variable	All modalities	CT	X-ray	MRI	Ultrasound
Imaging not performed	88 (18.4)	194 (40.5)	301 (62.8)	454 (94.8)	322 (67.2)
Imaging performed	391 (81.6)	285 (59.5)	178 (37.2)	25 (5.2)	157 (32.8)
Foreign body not seen on imaging	203 (42.4)	129 (26.9)	165 (34.4)	21 (4.4)	112 (23.4)
Foreign body seen on imaging	188 (39.2)	156 (32.6)	13 (2.7)	4 (0.8)	45 (9.4)

Modalities evaluated include computed tomography (CT), X-ray, magnetic resonance imaging (MRI), and ultrasound. Data are expressed in number of the cases (percentage).

### Endoscopic management

Endoscopic procedures were performed in 222 cases (46.3%) and the foreign body was directly visualized on endoscopy in 76.1% (169/222) of cases (Table 6). Among cases in which the foreign body was visualized, perforation was noted in 74.6% of cases. The foreign body was successfully removed in 88.8% of cases (150/169). The tools used to remove the foreign body are summarized in Supplementary Table S12. Clips were not placed in 128 cases after removal, with a low rate (2.3%, 3/128) of subsequent adverse events such as bleeding or infection. After endoscopic removal of the foreign body, 4.7% (7/150) of patients required subsequent surgery, for bleeding, infection, or small bowel obstruction. Of the 11.2% of cases (19/169) in which the object was not removed endoscopically, most were not removed due to operator discomfort with doing so. There was one case in which the object was unable to be removed due to patient discomfort when touching the object and two cases in which the object was unable to be dislodged.

**Table 6.** Summary of endoscopic procedures performed, removal, and outcomes

Variable	*n* (%)
No procedure performed	257 (53.7)
Procedure performed	222 (46.3)
Foreign body not seen on procedure	53 (11.1)
Foreign body seen on procedure	169 (35.3)
Average number of endoscopic procedures performed before diagnosis	1.1
Types of procedures performed^a^	
Upper endoscopy	148 (66.7)
Push enteroscopy	2 (0.9)
Single balloon	2 (0.9)
Double balloon	3 (1.4)
Colonoscopy	61 (27.5)
Video capsule endoscopy	3 (1.4)
Endoscopic ultrasound	14 (6.3)
Endoscopic retrograde cholangiopancreatography	4 (1.8)
Sigmoidoscopy	13 (5.9)
Anoscopy	5 (2.3)
Foreign body removal^b^	
Foreign body seen but not removed	19 (11.2)
Foreign body seen and removed	150 (88.8)
Outcomes post foreign body removal	
Adverse events necessitating surgery^c^	7 (4.7)
Adverse events in cases using a clip^d^	2 (9.1)
Adverse events in cases without clipping^e^	3 (2.3)

a Percentages are out of the 222 cases in which a procedure was performed.

b Percentages are out of the 169 cases in which the foreign body was seen.

c Percentages are out of the 150 cases in which the foreign body was removed.

d Percentages are out of the 22 cases in which clipping was performed.

e Percentages are out of the 128 cases in which clipping was not performed.

### Surgical management

Surgery was performed in 318 cases (66.4%), with a bowel resection performed in 90 of these cases (28.3%). Post-operative adverse events were rare and included bleeding (1.9%, 6/318) followed by equal occurrences of surgical wound infections, abscesses, post-operative fever, cardiopulmonary issues, or death (0.9%, 3/318). Of note, 3.5% of patients did require at least one follow-up surgery, with 0.3% of patients requiring an endoscopic procedure (bile leak requiring stenting).

### Other outcomes

Two hundred and twenty cases (45.9%) reported administration of antibiotics. Overall, ingestion of sharp wooden objects resulted in death in 24 cases (5.0%).

### Stratified analysis by awareness of ingestion

To explore whether outcomes and/or clinical management differed based on whether the patient was aware of their ingestion at the time of presentation, we performed a stratified analysis based on awareness of ingestion at time of presentation.

Patients who were unaware of any ingestion tended to be slightly older and more likely to have substance-use disorder or to be edentulous compared with those who were aware (Table 7). There was no significant difference in terms of complications related to a delay in diagnosis between the two groups. The foreign bodies were primarily located in the GI tract in both groups, but more so in aware patients (88.4% vs 77.8%). Rates of endoscopic removal were similar in the two groups. A significantly higher proportion of unaware patients at the time of presentation ultimately required surgery (66.7% vs 45.5%, *P *=* *0.01). Among aware patients, the time from ingestion to evaluation varied considerably, with a median of 7 days and an interquartile range of 41 days.

**Table 7.** Patient demographics and details of sharp wooden object ingestions including outcomes stratified by awareness at the time of presentation

Variable	No awareness		Awareness		*P*-value
	Number of cases^a^	Results (%)	Number of cases^a^	Results (%)	
Age, years, median (IQR)	241	55 (44, 65)	43	38 (26, 52)	<0.01
Sex	242		44		0.23
Male		165 (68.2)		34 (77.3)	
Complication related to delay	243		44		0.24
Yes		32 (13.2)		3 (6.8)	
Determined location of object	243		43		0.02
Gastrointestinal tract		189 (77.8)		38 (88.4)	
Esophagus		5 (2.1)		1 (2.3)	
Stomach		39 (16.0)		14 (32.6)	
Small bowel		71 (29.2)		12 (27.9)	
Duodenum		28 (11.5)		11 (25.6)	
Jejunum		12 (4.9)		0 (0)	
Ileum		24 (9.9)		0 (0)	
Unspecified small bowel location		7 (2.9)		1 (2.3)	
Ileocecal valve		14 (5.8)		0 (0)	
Appendix		4 (1.6)		0 (0)	
Colorectal		64 (26.3)		14 (32.6)	
Colon		58 (23.9)		14 (32.6)	
Rectum		6 (2.5)		0 (0)	
Liver		23 (9.5)		4 (9.3)	
Other		86 (35.4)		11 (25.6)	
Object endoscopically removed	109		28		0.26
No		31 (28.4)		5 (17.9)	
Not seen		23 (21.1)		3 (10.7)	
Seen, not removed		8 (7.3)		2 (7.1)	
Yes		78 (71.6)		23 (82.1)	
Surgery required	243		44		0.01
Yes		162 (66.7)		20 (45.5)	
Mortality	243		44		0.20
Deaths		9 (3.7)		0 (0)	

IQR = interquartile range.

a Reflects the number of cases for which the data were reported.

### Stratified analysis by use of endoscopy

To investigate the factors that were associated with the decision to pursue endoscopy, we performed a stratified analysis based on performance of endoscopy. We found no significant differences in terms of age, sex, race/ethnicity, or comorbidities between patients who did or did not undergo endoscopy (Table 8). Endoscopy was performed more often in patients who were aware of their ingestion (20.4% vs 10.7%, *P *=* *0.01) and imaging showed an intraluminal/transluminal object (79.6% vs 56.8%, *P *<* *0.001). Patients who presented with gastrointestinal bleeding were more likely to undergo endoscopy (17.6% vs 3.5%, *P *<* *0.001). We also found that the yield of endoscopic visualization correlated with imaging findings: 90.4% (47/52) for intraluminal/transluminal objects but only 18.2% (2/11) for extraluminal objects on imaging. In aware patients who underwent endoscopy with negative imaging findings, 100% of objects that involved the GI tract (stomach, duodenum, or colon only) were still found, with 90% (9/10) seen endoscopically.

**Table 8.** Patient demographics and details of sharp wooden object ingestions stratified by use of endoscopy

Variable	No endoscopy performed		Endoscopy performed		*P*-value
	Number of cases^a^	Results (%)	Number of cases^a^	Results (%)	
Age, years, median (IQR)	253	55 (42, 64)	220	53 (42, 64)	0.84
Sex	256		219		0.13
Male		187 (73.0)		146 (66.7)	
Knowledge of ingestion	150		137		0.01
Aware		16 (10.7)		28 (20.4)	
Unaware		134 (89.3)		109 (79.6)	
Seen on imaging	198		184		0.57
Yes		90 (45.5)		89 (48.4)	
Location on imaging	95		93		<0.01
Transluminal/intraluminal		54 (56.8)		74 (79.6)	
Extraluminal		41 (43.2)		19 (20.4)	
Adverse events	257		222		<0.01
No adverse events		2 (0.8)		29 (13.1)	
Gastrointestinal bleeding		9 (3.5)		39 (17.6)	
Abscess		89 (34.6)		69 (31.1)	
Bacteremia		20 (7.8)		20 (9.0)	
Perforation		242 (94.2)		177 (79.7)	
Other		86 (33.5)		68 (30.6)	

IQR = interquartile range.

a Reflects the number of cases for which the data were reported.

## Discussion

The findings from this large systematic review highlight the diverse clinical presentations and management approaches for the ingestion of sharp wooden objects such as toothpicks.

Compared with previous retrospective studies [18] and systematic reviews [19], our study noted different frequencies of organ involvement and provided a more comprehensive overview of other affected organs, likely due to the larger number of cases assembled. While the GI tract was the most frequent location in which the objects were found, there was also significant involvement of other organs such as the liver (11.9%) and vascular structures including the iliac arteries (2.7%) and inferior vena cava (1.7%). The higher observed incidence of right-sided intra-abdominal involvement likely reflects the angulation and luminal narrowing at sites such as the pylorus, duodenal sweep, ileocecal valve, and hepatic flexure, which can impede the passage of sharp objects. Certain object characteristics such as size, shape, and rigidity may also predispose impaction at these anatomic bottlenecks [20, 21].

We found that most patients who have been reported to have sharp wooden object ingestions were middle-aged men, with substance-use disorders and edentulousness being notable risk factors. The observed associations with substance-use disorder and edentulousness provide insights into potential mechanisms that increase ingestion risk. Substance use can lead to impaired judgment, lack of coordination, and decreased awareness―all factors that may contribute to unintentional ingestions. Edentulousness may be a marker for poor oral hygiene practices and may predispose to accidental ingestion.

Lack of awareness regarding the ingestion event was very common. This is important, as our stratified analysis revealed that patients who were unaware of ingestion were more likely to have extraluminal migration of the foreign body, have adverse events, require surgical intervention, and die compared with aware patients. Similarly, in adjusted regression analysis, we found that awareness of ingestion was associated with 69% decreased odds of developing an adverse event, likely because awareness of ingestion leads to more expedient presentation and development. Our finding that endoscopy was also associated with significantly decreased odds of adverse events related to sharp wooden object ingestion likely reflects the early recognition and removal of the object. Overall, our findings underscore the importance of early recognition, as delayed presentations allow more time for transmural passage and complications. Intentional ingestions and those with a clear ingestion history should prompt expedited evaluation and management.

Imaging was frequently performed and our study confirms the general sentiment that CT scans are helpful for the identification of foreign bodies and perforation—particularly those that are not radiopaque [22–24]. In our study, we found that CT has a higher sensitivity for toothpicks than previous studies mentioned earlier (54.7% compared with 42.6% [18] or 15.0% [19]). Even if the foreign body is not well visualized on imaging, signs of perforation such as free air, bowel wall thickening, or focal bowel wall discontinuity may be seen, especially with the use of intravenous contrast, which should raise the suspicion of foreign body-related injury [25]. In addition, endoscopy had a high yield if the object was noted to be intraluminal or transluminal in reachable areas on imaging and frequently had a high yield in patients with negative imaging—particularly those who were aware of their ingestion.

As reported in previous studies [26–28], we also found that endoscopic removal of sharp wooden objects was safe and efficacious. While we reported the various tools used for endoscopic retrieval, the exact choice for management should be determined by factors including the object size, shape, orientation, and location. Timing of the intervention is also crucial, as very early ingestions may be amenable to simpler techniques such as retrieval from the stomach, whereas longer delays may require more advanced methods such as deep enteroscopy or the use of additional tools for the removal of partially transmigrated objects.

The 5% mortality rate observed in our systematic review highlights the potential clinical severity of such ingestions. Older age, comorbidities, delays in diagnosis, and complications such as perforation likely contributed to mortality. Long-term follow-up data on morbidities such as fistulae, abscesses, and strictures were limited but they are important to understand, given their profound impact on quality of life. Based on the risk factors identified, targeted prevention strategies are needed. Public awareness campaigns about the dangers of ingesting non-food items, better packaging for toothpicks/skewers, and increased supervision of high-risk groups such as those with substance abuse or cognitive impairment may help to reduce the burden of these ingestions. Regulatory policies around the packaging and labeling of such products could also be considered.

**Figure 2.**
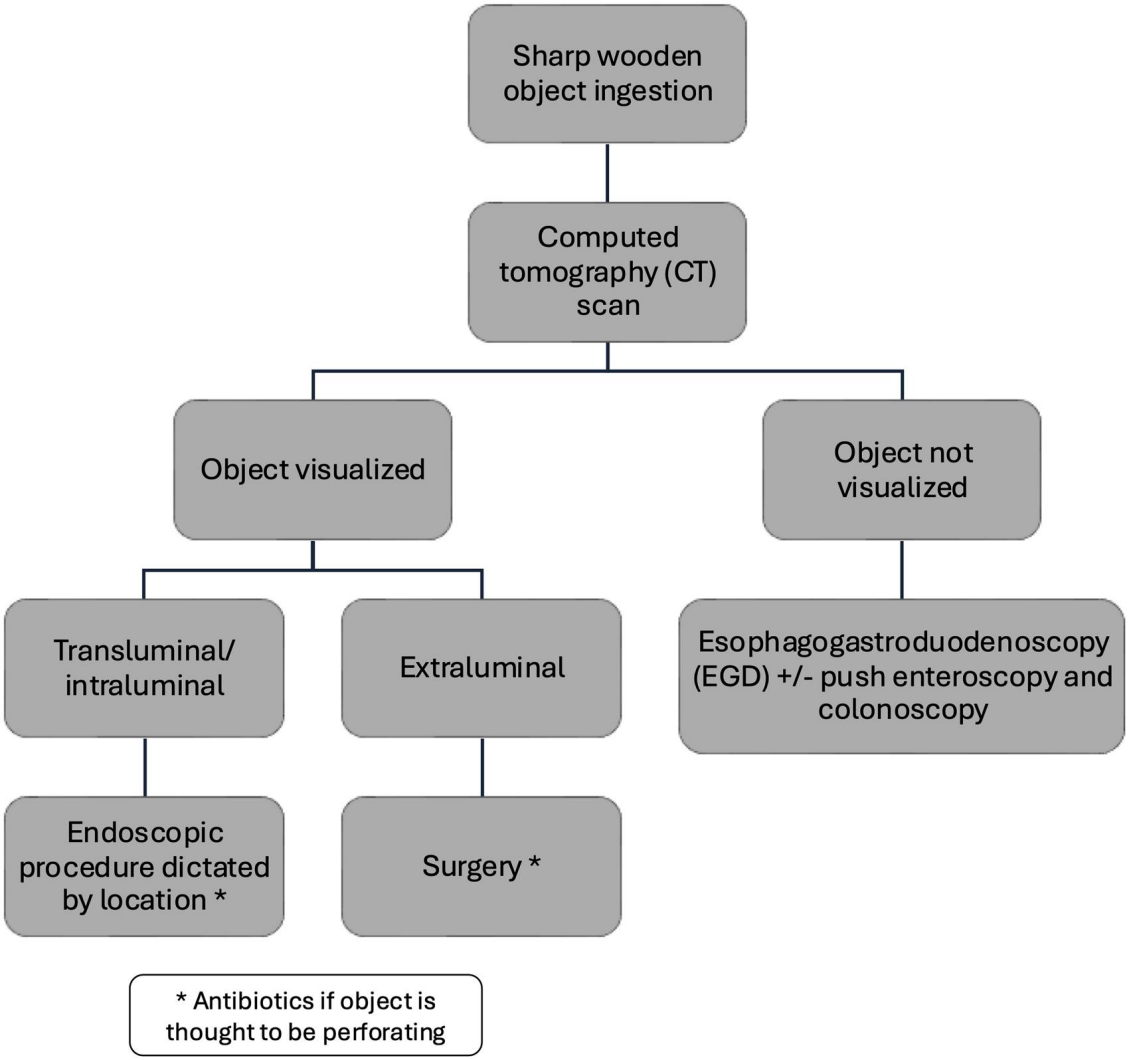
Proposed clinical algorithm for managing a confirmed case of sharp wooden object ingestion.

Given the results of our analysis, we propose a clinical algorithm for managing patients with suspected or known sharp wooden object ingestion. In patients presenting with concern for sharp wooden object ingestion, a CT with intravenous contrast is the most reasonable initial step for all patients. If the object is not seen on CT, then an upper endoscopy with or without push enteroscopy should be performed to evaluate the proximal GI tract regardless of the time course of ingestion or current symptoms. If the object is visualized within the GI tract on CT in a transluminal or intraluminal location, then endoscopic evaluation should be performed, with the endoscopic modality determined based on location. For objects visualized on CT in an extraluminal location, surgical management is appropriate (Figure 2).

If the object is seen endoscopically, then it should be removed with or without mechanical clipping. If the foreign body is not seen endoscopically, then the next steps such as surgery or further imaging with repeat CT or ultrasound can be considered, depending on the patient’s clinical condition. Antibiotics should be strongly considered for all patients with evidence of perforation, based on imaging or endoscopic findings.

There are several inherent limitations to this study. There is unavoidable selection bias, as the individual patient data were obtained from case reports and case series. There are also missing data from some studies, such as timing after ingestion to endoscopy. Reporting bias is also likely present. Given that the data were extracted from studies from 1911 to the present, there may also be an era effect due to advances in imaging, diagnostic and therapeutic endoscopy, and surgical techniques. While we acknowledge the limitations that are inherent in the synthesis of case reports and case series, in the absence of higher levels of evidence (e.g. randomized clinical trials, observational cohort studies), such syntheses have been important to advance medical knowledge and provide information for clinical situations in which there would otherwise be no evidence base to guide best practices [14]. Finally, an additional limitation arises from the variability in descriptions and potential scarcity of mentions of toothpicks in the titles, abstracts, and other indexed fields within the databases. To mitigate this limitation, we undertook a rigorous approach of citation chasing, reviewing many with unclear descriptions of the foreign bodies in full text.

## Conclusions

Ingestion of a toothpick or similar sharp wooden object is often difficult to diagnose. It should be suspected in someone with unexplained abdominal pain, especially in the presence of GI bleeding, infection, mass, or fistula. Through our algorithm for approaching suspected sharp wooden object ingestion, we hope to provide gastroenterologists and other acute care physicians with an evidence-based paradigm to evaluate and manage such patients.

## Supplementary Material

goaf035_Supplementary_Data

## References

[1] Riddle PJ , Mujadzic H, Noorani S, Peluso H, Abougergi M. S579 Foreign Body Ingestion: a Hard Pill to Swallow. Am J Gastroenterol 2021;116:S263–4.

[2] Velitchkov NG , Grigorov GI, Losanoff JE et al Ingested foreign bodies of the gastrointestinal tract: retrospective analysis of 542 cases. World J Surg 1996;20:1001–5. 8798356 10.1007/s002689900152

[3] Smith MT , Wong RK. Foreign bodies. Gastrointest Endosc Clin N Am 2007;17:361–82, vii. 17556153 10.1016/j.giec.2007.03.002

[4] Goh BKP , Chow PKH, Quah H-M et al Perforation of the gastrointestinal tract secondary to ingestion of foreign bodies. World J Surg 2006;30:372–7. 16479337 10.1007/s00268-005-0490-2

[5] Schoffstall JM , McNamara RM. More on ingestion of a toothpick. N Engl J Med 1989;321:476–7. 2761584

[6] Barros JL , Caballero Jr. A, Rueda JC et al Foreign body ingestion: management of 167 cases. World J Surg 1991;15:783–8. 1767546 10.1007/BF01665320

[7] Katsinelos P , Kountouras J, Paroutoglou G et al Endoscopic techniques and management of foreign body ingestion and food bolus impaction in the upper gastrointestinal tract: a retrospective analysis of 139 cases. J Clin Gastroenterol 2006;40:784–9. 17016132 10.1097/01.mcg.0000225602.25858.2c

[8] Zhang S , Cui Y, Gong X et al Endoscopic management of foreign bodies in the upper gastrointestinal tract in South China: a retrospective study of 561 cases. Dig Dis Sci 2010;55:1305–12. 19655249 10.1007/s10620-009-0900-7

[9] Page MJ , McKenzie JE, Bossuyt PM et al The PRISMA 2020 statement: an updated guideline for reporting systematic reviews. BMJ 2021;372:n71. 33782057 10.1136/bmj.n71 PMC8005924

[10] Campbell M , McKenzie JE, Sowden A et al Synthesis without meta-analysis (SWiM) in systematic reviews: reporting guideline. BMJ 2020;368:l6890. 31948937 10.1136/bmj.l6890 PMC7190266

[11] McGowan J , Sampson M, Salzwedel DM et al PRESS peer review of electronic search strategies: 2015 guideline statement. J Clin Epidemiol 2016;75:40–6. 27005575 10.1016/j.jclinepi.2016.01.021

[12] Haddaway NR , Grainger MJ, Gray CT, citationchaser: an R package for forward and backward citations chasing in academic searching. 0.0.3 ed. 2021. https://cran.rstudio.com/web/packages/citationchaser/ (5 January 2024, date last accessed).

[13] Yale University Harvey Cushing/John Hay Whitney Medical Library. Reference Deduplicator. 2021. https://library.medicine.yale.edu/reference-deduplicator.

[14] Murad MH , Sultan S, Haffar S et al Methodological quality and synthesis of case series and case reports. BMJ Evid Based Med 2018;23:60–3. 10.1136/bmjebm-2017-110853 PMC6234235 29420178

[15] Rozner R , Gisriel S, Damianos J et al Idiopathic myointimal hyperplasia of the mesenteric veins: A systematic review and individual patient data regression analysis. J Gastroenterol Hepatol 2023;38:1040–6. 37086041 10.1111/jgh.16193

[16] Lam R , Tarangelo N, Wang R et al Microangiopathic hemolytic anemia is a late and fatal complication of gastric signet ring cell carcinoma: a systematic review and case-control study. Oncologist 2022;27:751–9. 35589098 10.1093/oncolo/oyac093 PMC9438916

[17] Li DK , Haffar S, Horibe M et al Verrucous esophageal carcinoma is a unique indolent subtype of squamous cell carcinoma: a systematic review and individual patient regression analysis. J Gastroenterol 2021;56:12–24. 33079233 10.1007/s00535-020-01736-1

[18] Steinbach C , Stockmann M, Jara M et al Accidentally ingested toothpicks causing severe gastrointestinal injury: a practical guideline for diagnosis and therapy based on 136 case reports. World J Surg 2014;38:371–7. 24166027 10.1007/s00268-013-2307-z

[19] Li SF , Ender K. Toothpick injury mimicking renal colic: case report and systematic review. J Emerg Med 2002;23:35–8. 12217469 10.1016/s0736-4679(02)00458-4

[20] Ginsberg GG. Management of ingested foreign objects and food bolus impactions. Gastrointest Endosc 1995;41:33–8. 7698622 10.1016/s0016-5107(95)70273-3

[21] Wozniak S , Pytrus T, Kobierzycki C et al The large intestine from fetal period to adulthood and its impact on the course of colonoscopy. Ann Anat 2019;224:17–22. 30914345 10.1016/j.aanat.2019.02.004

[22] Takada M , Kashiwagi R, Sakane M et al 3D-CT diagnosis for ingested foreign bodies. Am J Emerg Med 2000;18:192–3. 10750930 10.1016/s0735-6757(00)90018-4

[23] Marco De Lucas E , Sádaba P, Lastra García-Barón P et al Value of helical computed tomography in the management of upper esophageal foreign bodies. Acta Radiol 2004;45:369–74. 15323387 10.1080/02841850410005516

[24] Guelfguat M , Kaplinskiy V, Reddy SH et al Clinical guidelines for imaging and reporting ingested foreign bodies. AJR Am J Roentgenol 2014;203:37–53. 24951194 10.2214/AJR.13.12185

[25] Simonetti I , Puglia M, Tarotto L et al When traditions become dangerous: Intestinal perforation from unusual foreign body-Case report and short literature review. Eur J Radiol Open 2019;6:152–5. 31024984 10.1016/j.ejro.2019.04.002 PMC6475829

[26] Li G , Wu D, Zhou L et al Delayed endoscopic management of esophageal sharp-pointed food impaction: an analysis of 829 cases in China. Dig Dis Sci 2022;67:3166–76. 34342753 10.1007/s10620-021-07133-9

[27] Liao F , Zhu Z, Pan X et al Safety and efficacy of nonoperative treatment in esophageal perforation caused by foreign bodies. Clin Transl Gastroenterol 2022;13:e00451. 35060929 10.14309/ctg.0000000000000451 PMC8806378

[28] Ugenti I , Digennaro R, Martines G et al Double esophageal perforation by ingested foreign body: endoscopic and surgical approach. A case report. Int J Surg Case Rep 2015;17:55–7. 26551553 10.1016/j.ijscr.2015.10.033 PMC4701816

