# Supplementary material for: Clinical features and endoscopic management of sharp wooden object ingestions: a systematic review of 479 cases
Source: Gastroenterol Rep (Oxf). 2025 May 14;13:goaf035. doi: 10.1093/gastro/goaf035 (PMC12073997; doi:10.1093/gastro/goaf035)
Supplement: goaf035_Supplementary_Data [file goaf035_supplementary_data.zip › Supplementary Material 20250408.docx]

**Supplementary Material**

**Supplementary Table 1**. PRISMA Checklist

| Topic | No. | Item | Location where item is reported |
| --- | --- | --- | --- |
| TITLE |  |  |  |
| Title | 1 | Identify the report as a systematic review. | LN2–3 |
| ABSTRACT |  |  |  |
| Abstract | 2 | See the PRISMA 2020 for Abstracts checklist | Suppl. 1 |
| INTRODUCTION |  |  |  |
| Rationale | 3 | Describe the rationale for the review in the context of existing knowledge. | LN48–53 |
| Objectives | 4 | Provide an explicit statement of the objective(s) or question(s) the review addresses. | LN54–62 |
| METHODS |  |  |  |
| Eligibility criteria | 5 | Specify the inclusion and exclusion criteria for the review and how studies were grouped for the syntheses. | LN71–73 |
| Information sources | 6 | Specify all databases, registers, websites, organisations, reference lists and other sources searched or consulted to identify studies. Specify the date when each source was last searched or consulted. | LN76–82 |
| Search strategy | 7 | Present the full search strategies for all databases, registers and websites, including any filters and limits used. | Suppl. Table 4 |
| Selection process | 8 | Specify the methods used to decide whether a study met the inclusion criteria of the review, including how many reviewers screened each record and each report retrieved, whether they worked independently, and if applicable, details of automation tools used in the process. | LN85–90 |
| Data collection process | 9 | Specify the methods used to collect data from reports, including how many reviewers collected data from each report, whether they worked independently, any processes for obtaining or confirming data from study investigators, and if applicable, details of automation tools used in the process. | LN87–89 |
| Data items | 10a | List and define all outcomes for which data were sought. Specify whether all results that were compatible with each outcome domain in each study were sought (e.g. for all measures, time points, analyses), and if not, the methods used to decide which results to collect. | LN93–98 |
|  | 10b | List and define all other variables for which data were sought (e.g. participant and intervention characteristics, funding sources). Describe any assumptions made about any missing or unclear information. | LN93–98 |
| Study risk of bias assessment | 11 | Specify the methods used to assess risk of bias in the included studies, including details of the tool(s) used, how many reviewers assessed each study and whether they worked independently, and if applicable, details of automation tools used in the process. | LN107–109 |
| Effect measures | 12 | Specify for each outcome the effect measure(s) (e.g. risk ratio, mean difference) used in the synthesis or presentation of results. | LN112–128 |
| Synthesis methods | 13a | Describe the processes used to decide which studies were eligible for each synthesis (e.g. tabulating the study intervention characteristics and comparing against the planned groups for each synthesis (item 5)). | LN120–128 |
|  | 13b | Describe any methods required to prepare the data for presentation or synthesis, such as handling of missing summary statistics, or data conversions. | LN120–128 |
|  | 13c | Describe any methods used to tabulate or visually display results of individual studies and syntheses. | LN120–128 |
|  | 13d | Describe any methods used to synthesize results and provide a rationale for the choice(s). If meta-analysis was performed, describe the model(s), method(s) to identify the presence and extent of statistical heterogeneity, and software package(s) used. | LN120–128 |
|  | 13e | Describe any methods used to explore possible causes of heterogeneity among study results (e.g. subgroup analysis, meta-regression). | N/A |
|  | 13f | Describe any sensitivity analyses conducted to assess robustness of the synthesized results. | N/A |
| Reporting bias assessment | 14 | Describe any methods used to assess risk of bias due to missing results in a synthesis (arising from reporting biases). | LN107–109 |
| Certainty assessment | 15 | Describe any methods used to assess certainty (or confidence) in the body of evidence for an outcome. | N/A |
| RESULTS |  |  |  |
| Study selection | 16a | Describe the results of the search and selection process, from the number of records identified in the search to the number of studies included in the review, ideally using a flow diagram. | LN132–142 |
|  | 16b | Cite studies that might appear to meet the inclusion criteria, but which were excluded, and explain why they were excluded. | Suppl. Table 7 |
| Study characteristics | 17 | Cite each included study and present its characteristics. | Suppl. Table 8–9 |
| Risk of bias in studies | 18 | Present assessments of risk of bias for each included study. | LN145–148 |
| Results of individual studies | 19 | For all outcomes, present, for each study: (a) summary statistics for each group (where appropriate) and (b) an effect estimate and its precision (e.g. confidence/credible interval), ideally using structured tables or plots. | LN151–156 |
| Results of syntheses | 20a | For each synthesis, briefly summarise the characteristics and risk of bias among contributing studies. | LN151–269 |
|  | 20b | Present results of all statistical syntheses conducted. If meta-analysis was done, present for each the summary estimate and its precision (e.g. confidence/credible interval) and measures of statistical heterogeneity. If comparing groups, describe the direction of the effect. | N/A |
|  | 20c | Present results of all investigations of possible causes of heterogeneity among study results. | N/A |
|  | 20d | Present results of all sensitivity analyses conducted to assess the robustness of the synthesized results. | N/A |
| Reporting biases | 21 | Present assessments of risk of bias due to missing results (arising from reporting biases) for each synthesis assessed. | LN145–148 |
| Certainty of evidence | 22 | Present assessments of certainty (or confidence) in the body of evidence for each outcome assessed. | LN145–148 |
| DISCUSSION |  |  |  |
| Discussion | 23a | Provide a general interpretation of the results in the context of other evidence. | LN272–341 |
|  | 23b | Discuss any limitations of the evidence included in the review. | LN342–354 |
|  | 23c | Discuss any limitations of the review processes used. | LN342–354 |
|  | 23d | Discuss implications of the results for practice, policy, and future research. | LN357–361 |
| OTHER INFORMATION |  |  |  |
| Registration and protocol | 24a | Provide registration information for the review, including register name and registration number, or state that the review was not registered. | LN65–66 |
|  | 24b | Indicate where the review protocol can be accessed, or state that a protocol was not prepared. | LN65–66 |
|  | 24c | Describe and explain any amendments to information provided at registration or in the protocol. | N/A |
| Support | 25 | Describe sources of financial or non-financial support for the review, and the role of the funders or sponsors in the review. | LN20 |
| Competing interests | 26 | Declare any competing interests of review authors. | LN21–22 |
| Availability of data, code and other materials | 27 | Report which of the following are publicly available and where they can be found: template data collection forms; data extracted from included studies; data used for all analyses; analytic code; any other materials used in the review. | Upon Request |

Adapted from: Page MJ, McKenzie JE, Bossuyt PM, Boutron I, Hoffmann TC, Mulrow CD, *et al*. The PRISMA 2020 statement: an updated guideline for reporting systematic reviews. MetaArXiv. 2020, September 14. DOI: 10.31222/osf.io/v7gm2.

**Supplementary Table 2**. PRISMA Abstract Checklist.

| Topic | No. | Item | Reported? |
| --- | --- | --- | --- |
| TITLE |  |  |  |
| Title | 1 | Identify the report as a systematic review. | Yes |
| BACKGROUND |  |  |  |
| Objectives | 2 | Provide an explicit statement of the main objective(s) or question(s) the review addresses. | Yes |
| METHODS |  |  |  |
| Eligibility criteria | 3 | Specify the inclusion and exclusion criteria for the review. | Yes |
| Information sources | 4 | Specify the information sources (e.g. databases, registers) used to identify studies and the date when each was last searched. | Yes |
| Risk of bias | 5 | Specify the methods used to assess risk of bias in the included studies. | Yes |
| Synthesis of results | 6 | Specify the methods used to present and synthesize results. | Yes |
| RESULTS |  |  |  |
| Included studies | 7 | Give the total number of included studies and participants and summarise relevant characteristics of studies. | Yes |
| Synthesis of results | 8 | Present results for main outcomes, preferably indicating the number of included studies and participants for each. If meta-analysis was done, report the summary estimate and confidence/credible interval. If comparing groups, indicate the direction of the effect (i.e. which group is favoured). | Yes |
| DISCUSSION |  |  |  |
| Limitations of evidence | 9 | Provide a brief summary of the limitations of the evidence included in the review (e.g. study risk of bias, inconsistency and imprecision). | Yes |
| Interpretation | 10 | Provide a general interpretation of the results and important implications. | Yes |
| OTHER |  |  |  |
| Funding | 11 | Specify the primary source of funding for the review. | Yes |
| Registration | 12 | Provide the register name and registration number. | Yes |

The citation for the Synthesis Without Meta-analysis explanation and elaboration article is: Campbell M, McKenzie JE, Sowden A, Katikireddi SV, Brennan SE, Ellis S, Hartmann-Boyce J, Ryan R, Shepperd S, Thomas J, Welch V, Thomson H. Synthesis without meta-analysis (SWiM) in systematic reviews: reporting guideline. BMJ. 2020;368:l6890.doi:10.1136/bmj.l6890

**Supplementary Table 3**. SWiM is intended to complement and be used as an extension to PRISMA.

| SWiM reporting item | Item description | Page in manuscript where item is reported |
| --- | --- | --- |
| *Methods* | | |
| 1 Grouping studies for synthesis | 1a) Provide a description of, and rationale for, the groups used in the synthesis (e.g., groupings of populations, interventions, outcomes, study design) | 7–8 |
|  | 1b) Detail and provide rationale for any changes made subsequent to the protocol in the groups used in the synthesis | N/A |
| 2 Describe the standardised metric and transformation methods used | Describe the standardised metric for each outcome. Explain why the metric(s) was chosen, and describe any methods used to transform the intervention effects, as reported in the study, to the standardised metric, citing any methodological guidance consulted | 7–8 |
| 3 Describe the synthesis methods | Describe and justify the methods used to synthesise the effects for each outcome when it was not possible to undertake a meta-analysis of effect estimates | 7–8 |
| 4 Criteria used to prioritise results for summary and synthesis | Where applicable, provide the criteria used, with supporting justification, to select the particular studies, or a particular study, for the main synthesis or to draw conclusions from the synthesis (e.g., based on study design, risk of bias assessments, directness in relation to the review question) | 7–8 |
| 5 Investigation of heterogeneity in reported effects | State the method(s) used to examine heterogeneity in reported effects when it was not possible to undertake a meta-analysis of effect estimates and its extensions to investigate heterogeneity | 7–8 |
| 6 Certainty of evidence | Describe the methods used to assess certainty of the synthesis findings | 7–8 |
| 7 Data presentation methods | Describe the graphical and tabular methods used to present the effects (e.g., tables, forest plots, harvest plots).  Specify key study characteristics (e.g., study design, risk of bias) used to order the studies, in the text and any tables or graphs, clearly referencing the studies included | 7–8 |
| *Results* | | |
| 8 Reporting results | For each comparison and outcome, provide a description of the synthesised findings, and the certainty of the findings. Describe the result in language that is consistent with the question the synthesis addresses, and indicate which studies contribute to the synthesis | 5–10 |
| *Discussion* | | |
| 9 Limitations of the synthesis | Report the limitations of the synthesis methods used and/or the groupings used in the synthesis, and how these affect the conclusions that can be drawn in relation to the original review question | 12–13 |

PRISMA, Preferred Reporting Items for Systematic Reviews and Meta-Analyses.

**Supplementary Table 4.** Search strategies.

| Database | Search strategies |
| --- | --- |
| Ovid Embase | 1 (toothpick* or tooth pick* or pick tooth* or chopstick* or chop stick* or bamboo pick* or wooden foreign bod* or dental pick*).tw,kw.  2 ((lollipop* or popsicle or sandwich or kebab* or doeurves or oeurves or appetizer* or food or wooden or cupcake* or dessert*) adj1 (stick or sticks or skewer* or spear* or pick or picks or sword*)).tw,kw.  3 ((meat or steak) adj1 (marker* or pick or picks or skewer* or stick or sticks or spear or spears)).tw,kw.  4 ((beverage* or cocktail* or drink*) adj1 (stick or sticks or skewer* or spear* or pick or picks or stirrer* or sword* or umbrella*)).tw,kw.  5 1 or 2 or 3 or 4  6 exp animal/  7 exp animal/ and exp human/  8 6 not 7  9 5 not 8 |
| Ovid MEDLINE(R) ALL | 1 (toothpick* or tooth pick* or pick tooth* or chopstick* or chop stick* or bamboo pick* or wooden foreign bod* or dental pick*).tw,kf.  2 ((lollipop* or popsicle or sandwich or kebab* or doeurves or oeurves or appetizer* or food or wooden or cupcake* or dessert*) adj1 (stick or sticks or skewer* or spear* or pick or picks or sword*)).tw,kf.  3 ((meat or steak) adj1 (marker* or pick or picks or skewer* or stick or sticks or spear or spears)).tw,kf.  4 ((beverage* or cocktail* or drink*) adj1 (stick or sticks or skewer* or spear* or pick or picks or stirrer* or sword* or umbrella*)).tw,kf.  5 1 or 2 or 3 or 4  6 exp animals/  7 exp animals/ and exp humans/  8 6 not 7  9 5 not 8 |
| Scopus | TITLE-ABS-KEY ( toothpick* OR tooth AND pick* OR pick AND tooth* OR chopstick* OR chop AND stick* OR bamboo AND pick* OR wooden AND foreign AND bod* OR dental AND pick* ) OR TITLE-ABS-KEY ( ( lollipop* OR popsicles OR sandwich OR kebab* OR doeuvres OR oeuvres OR appetizer* OR food OR wooden OR cupcake* OR dessert* ) W/1 ( stick OR sticks OR skewer* OR spear* OR pick OR picks OR sword* ) ) OR TITLE-ABS-KEY ( ( meat OR steak ) W/1 ( marker* OR pick OR picks OR skewer* OR stick OR sticks OR spear OR spears ) ) OR TITLE-ABS-KEY ( ( beverage* OR cocktail* OR drink* ) W/1 ( stick OR sticks OR skewer* OR spear* OR pick OR picks OR stirrer* OR sword* OR umbrella* ) ) |
| Web of Science Core Collection | #1 TS=(toothpick* or tooth pick* or pick tooth* or chopstick* or chop stick* or bamboo pick* or wooden foreign bod* or dental pick*) OR TS=((lollipop* or popsicles or sandwich or kebab* or doeuvres or oeuvres or appetizer* or food or wooden or cupcake* or dessert*) near/1 (stick or sticks or skewer* or spear* or pick or picks or sword*)) OR TS=((meat or steak) near/1 (marker* or pick or picks or skewer* or stick or sticks or spear or spears)) or TS=((beverage* or cocktail* or drink*) near/1 (stick or sticks or skewer* or spear* or pick or picks or stirrer* or sword* or umbrella*)) |
| PubMed | toothpick*[Title/Abstract] OR tooth pick*[Title/Abstract] OR pick tooth*[Title/Abstract] OR chopstick*[Title/Abstract] OR chop stick*[Title/Abstract] OR bamboo pick*[Title/Abstract] OR wooden foreign bod*[Title/Abstract] OR dental pick*[Title/Abstract] |
| Cochrane Library | (toothpick* or tooth pick* or pick tooth* or chopstick* or chop stick* or bamboo pick* or wooden foreign bod* or dental pick*):ti,ab OR ((lollipop* or popsicles or sandwich or kebab* or doeuvres or oeuvres or appetizer* or food or wooden or cupcake* or dessert*) near/1 (stick or sticks or skewer* or spear* or pick or picks or sword*)):ti,ab OR ((meat or steak) near/1 (marker* or pick or picks or skewer* or stick or sticks or spear or spears)):ti,ab or ((beverage* or cocktail* or drink*) near/1 (stick or sticks or skewer* or spear* or pick or picks or stirrer* or sword* or umbrella*)):ti,ab  Toothpick ingestion |
| Google Scholar |  |

**Supplementary Table 5.** Tool for assessment of the methodological quality of case reports and case series.

| Domains of quality assessment | Leading explanatory questions |
| --- | --- |
| Selection | Does the patient(s) represent(s) the entire experience of the researchers or is the selection modality unclear to the extent that other patients with similar presentation may have been missed? |
| Ascertainment | Was the outcome sufficiently ascertained? |
| Causality | Were other plausible causes that may account for the observation ruled out beyond a reasonable doubt? |
| Reporting | Is the case(s) relayed with adequate details to allow other investigators to replicate the research or to permit practitioners to make inferences related to their practice? |

Adapted from Murad MH, Sultan S, Haffar S, Bazerbachi F. Methodological quality and synthesis of case series and case reports. BMJ Evid Based Med. 2018;23(2):60-3. doi: 10.1136/bmjebm-2017-110853.

**Supplementary Table 6.** Papers unable to retrieved

| Item | Citation | Attempts to retrieve |
| --- | --- | --- |
| 1 | Buczynowska M, Banach A, Cienciala A, Topa J. Rare case of foreign body in the digestive tract. Proktologia 2006;7(3-4)():205-209. | Interlibrary loan, emailed last known email of authors, emailed publishers, and requested on Docline-L Listserv |
| 2 | Bonfiglio S, Cutuli S, Mazzarino C. A case of cecal perforation from foreign body. Rivista Italiana di Colon-Proctologia 1985;4(1)():74-76 | Interlibrary loan, emailed last known email of authors, emailed publishers, and requested on Docline-L Listserv |
| 3 | Sasaki K, Saito S, Miyajima A, Ueda Y, Ema A, Hirayama R, Otsuka R. RECURRENT LIVER ABSCESS CAUSED BY TOOTHPICK PENETRATION OF THE SIGMOID COLON. Gastroenterological Endoscopy 2023;65(3)():257-262. | Interlibrary loan, emailed last known email of authors, and requested on Docline-L Listserv |

**Supplementary Table 7.** Excluded studies.

| Surname of first author | Year | Title | Journal | Reason for exclusion |
| --- | --- | --- | --- | --- |
| Excluded studies from search | | | | |
| Abdalla | 2023 | Appendiceal Foreign Bodies in Adults: A Systematic Review of Case Reports | *Cureus* | Wrong study design |
| Ablett | 2009 | Appearance of wooden foreign body on CT scan | *Emergency Medicine Journal* | Wrong indication |
| Akkuzu | 2019 | Foreign bodies on the path of nutrition; retrospective evaluation of our clinical experience | *Turkish Journal of Gastroenterology* | Duplicate study data |
| Akkuzu | 2020 | Foreign bodies on the path of nutrition: Retrospective evaluation of our clinical experience. [Turkish] | *Haseki Tip Bulteni* | Wrong study design |
| Al-Addasi | 2021 | Toothpick perforation of the cecum in a child mimicking acute appendicitis | *Journal of Pediatric Surgery Case Reports* | Pediatric population |
| Alagiri | 1998 | Toothpick migration into bladder presents as abdominal pain and hematuria | *Urology* | Pediatric population |
| AlHarthy | 2022 | Superior Mesenteric Artery Pseudoaneurysm Induced by Accidental Ingestion of a Foreign Body: Case Report | *EJVES Vascular Forum* | Wrong indication |
| Badeaek-Izdebska | 2018 | Foreign bodies in the oesophagus on the example of patients of the Department of Paediatric Otolaryngology at the Medical University of Warsaw | *New Medicine* | Wrong patient population |
| Baer | 1986 | The dangerous toothpick | *The Journal of pedodontics* | Wrong study design |
| Barros | 1991 | Foreign body ingestion: management of 167 cases | *World J Surg* | Inadequate Information |
| Bauer | 2020 | [Dyspnoea Due to Foreign Embolism after Percutaneous Vertebroplasty] | *Pneumologie* | Wrong patient population |
| Bee | 1989 | MORE ON INGESTION OF A TOOTHPICK - REPLY | *New England Journal of Medicine* | Wrong study design |
| Berardino | 2008 | Laparoscopic surgery for intestinal perforation by foreign bodies | *Surgical Endoscopy* | Wrong study design |
| Bombizo | 2018 | From Interventional Radiology to Laparoscopic Liver Resection as Complementary Strategies in the Treatment of Hepatic Abscess Caused by Ingested Foreign Bodies | *Hepato-Gastroenterology* | Wrong indication |
| Bonaccorsi-Riani | 2012 | Toothpick-related injuries in the United States, 1979 through 1982 | *Journal of the American Medical Association* | Wrong study design |
| Budnick | 1984 | Toothpicks-related cardiovascular pathology. [French] | *Sang Thrombose Vaisseaux* | No original data |
| Carcone | 2019 | Treatment of patients with pyogenic liver abscess | *Chemotherapy* | Wrong study design |
| Cerwenka | 2005 | A stomach like a utility room: Case report | *Annals of Medicine and Surgery* | Wrong indication |
| Chen | 2021 | Clinical experience of ingested intraabdominal foreign bodies in children requiring surgical intervention | *Journal of Clinical Pediatric Surgery* | Wrong study design |
| Chen | 2021 | A case of gastric wall abscess caused by foreign body which successfully treated with endoscopic management | *Journal of Gastroenterology and Hepatology* | Inadequate Information |
| Cho | 2014 | An unusual case of duodenal perforation caused by a lollipop stick: a case report | *Clinical Endoscopy* | Wrong patient population |
| Cho | 2009 | The unusual cause of recurrent abdominal pain in an 11-year-old boy | *Nuclear Medicine Review* | Pediatric population |
| Chroustova | 2006 | Atypical fournier's gangrene: Gastrointestinal perforation associated with necrotizing fasciitis involving the abdominal wall, flank, or lower extremities | *Open Forum Infectious Diseases. Conference: ID Week* | Wrong indication |
| Cortes-Penfield | 2016 | A novel clip-assisted method for endoscopic removal of an impacted toothpick from the colon | *Endoscopy* | Pediatric population |
| DaMota | 2016 | Hypopharyngeal toothpick-induced granuloma mimicking a pyriform sinus malignancy | *Otolaryngology - Head and Neck Surgery* | Duplicate study data |
| Diggs | 2020 | Unexpected finding of an intra-peritoneal toothpick during laparoscopic appendectomy | *Journal of Pediatric Surgery Case Reports* | Pediatric population |
| ElMahmoud | 2019 | Concerning "Toothpicks: be careful!". [French] | *Journal de Chirurgie* | Wrong study design |
| Esquivel | 2015 | Pancreatic pseudotumor caused by toothpick ingestion | *Gastrointestinal Endoscopy* | Wrong study design |
| Faucher | 2001 | [Reason analysis of reoperation after failed laparoscopic surgery for acute abdomen] | *Zhonghua Wei Chang Wai Ke Za Zhi* | Wrong study design |
| Fu | 2012 | Successful removal of a toothpick penetrating the sigmoid colon by low-pressure endoscopy using the gel immersion method | *Endoscopy* | Pediatric population |
| Funayama | 2021 | Laparoscopic treatment of colorectal emergency | *Surgical Endoscopy and Other Interventional Techniques* | Wrong study design |
| Galleano | 2013 | Conservative Management of Duodenal Perforation with Toothpick in a 9-Year Old Girl; a Case Report | *International Journal of Pediatrics-Mashhad* | Pediatric population |
| Gheibi | 2016 | The diagnosis and treatment procedure in nonopaque foreign bodies in the stomach and intestines. [Russian] | *Klinicheskaia Khirurgiia* | Wrong study design |
| Gilenko | 1993 | Endoscopic removal of atypical foreign bodies from upper gastrointestinal tract: A single centre tertiary care experience from India | *American Journal of Gastroenterology* | Wrong study design |
| Goenka | 2021 | Perforation of the gastrointestinal tract secondary to ingestion of foreign bodies | *World Journal of Surgery* | Wrong study design |
| Goh | 2006 | Intestinal perforation due to an ingested foreign body: Laparoscopic management | *Journal of Laparoendoscopic Surgery* | Pediatric population |
| Hebra | 1996 | Toothpick-related injuries in the United States from 2001 to 2017 | *International journal of injury control and safety promotion* | Wrong study design |
| Hu | 2021 | 976 You Can't Always Pick Your Patients: Eus-Guided Transhepatic Removal of an Embedded Toothpick | *Gastrointestinal Endoscopy* | Duplicate study data |
| James | 2020 | Treatment considerations for cervical and cervicothoracic spondylodiscitis associated with esophageal fistula due to cancer history or accidental injury: a 9-patient case series | *Acta Neurochirurgica* | Inadequate Information |
| Janssen | 2019 | Intestinal perforation in adults due to ingested opaque foreign bodies | *American Journal of Gastroenterology* | Wrong patient population |
| Jr | 1976 | Meckel's diverticulum perforation by a wooden toothpick in a child: A case report | *Journal of Pediatric Surgery Case Reports* | Pediatric population |
| Kadhi | 2020 | Endoscopic techniques and management of foreign body ingestion and food bolus impaction in the upper gastrointestinal tract: A retrospective analysis of 139 cases | *J Clin Gastroenterol* | Wrong study design |
| Katsinelos | 2006 | Anal diseases from ingested foreign bodies. [Korean] | *Journal of the Korean Society of Coloproctology* | Inadequate Information |
| Khan | 2020 | Accidental ingestion of tooth pick leading to bowel perforation-A case report | *Pakistan Journal of Surgery* | Pediatric population |
| Kim | 2009 | An unusual cause of duodenal perforation due to a lollipop stick | *Korean Journal of Pediatrics* | Pediatric population |
| Kim | 2013 | Intestinal perforation by ingested foreign bodies | *Acta Medica Nagasakiensia* | Inadequate Information |
| Kinoshita | 2010 | Abdominal pain and hematuria: duodenal perforation from ingested foreign body causing ureteral obstruction and hydronephrosis | *J Surg Case Rep* | Wrong indication |
| Kolbe | 2016 | Foreign body ingestion mimicking irritable bowel syndrome: a case report | *J Med Case Rep* | Wrong patient population |
| Komninos | 2010 | Management of foreign bodies of the rectum: Report of 21 cases | *J. R. Coll. Surg. Edinb.* | Wrong patient population |
| Kouraklis | 1997 | Foreign body ingestion in children-five years of experience after the adoption of guidelines | *Archives of Disease in Childhood* | Wrong study design |
| Kovacic | 2021 | Letter to the Editor: Migration of a Toothpick between the Ascending Colon and Abdominal Wall Causing an Abdominal Mass | *Surgical infections* | Pediatric population |
| Kuang | 2022 | Foreign body ingestion in children-a 15-year retrospective analysis from a single academic center | *Journal of Pediatric Gastroenterology and Nutrition* | Wrong study design |
| Kumar | 2016 | A Rare Case Report of Severe Isolated Pre Pyloric Stenosis Developed Forty Days after Accidental Lye Ingestion: 1580 | *American Journal of Gastroenterology* | Wrong setting |
| Kurin | 2009 | Repeated long esophageal foreign bodies ingestion: A case report | *Journal of Internal Medicine of Taiwan* | Wrong patient population |
| Laass | 2019 | A Case of Wooden Foreign-Body Ingestion | *Journal of Emergency Medicine* | Wrong indication |
| Lanthaler | 2003 | Thigh cellulitis caused by toothpick ingestion | *Intensive Care Medicine* | Duplicate study data |
| Lee | 2009 | DETECTION OF WOODEN FOREIGN-BODIES | *Infections in Surgery* | Wrong patient population |
| Lee | 2018 | Acute complicated appendicitis caused by an ingested toothpick-A Case Report | *Surgical Endoscopy* | Duplicate study data |
| Lellouche | 2003 | FOREIGN-BODIES OF THE GASTROINTESTINAL-TRACT | *Medical Clinics of North America* | Wrong study design |
| Leonard | 1990 | Martini toothpick warning | *The New England journal of medicine* | Wrong study design |
| Lloyd | 2022 | Iliaco-coecal fistula caused by a penetrating toothpick. [German] | *Vasa - Journal of Vascular Diseases* | Duplicate study data |
| Lloyd | 2022 | Acute complicated appendicitis caused by an ingested toothpick - A case report | *International Journal of Surgery Case Reports* | Wrong patient population |
| Lyons | 1993 | Problems of diagnosis and treatment caused by ingested foreign bodies | *Chirurgia (Romania)* | Duplicate study data |
| Malamud | 1986 | Gastrointestinal perforations by ingested foreign bodies: A preoperative diagnostic flowchart-based experience. A case series report | *Int J Surg Case Rep* | Wrong study design |
| Mark | 1985 | Massive hemoptysis after aspiration of a toothpick | *Annals of Thoracic Surgery* | Wrong indication |
| Meaeina | 2013 | A retained wooden foreign body in the lung parenchyma presenting as aspergiloma | *Iranian Journal of Radiology* | Wrong patient population |
| Meșină | 2013 | Problems of diagnosis and treatment caused by ingested foreign bodies | *Chirurgia* | Duplicate study data |
| Mejri | 2022 | 'POOR OLD MR. TOOTHPICK' | *Black American Literature Forum* | Wrong study design |
| Misra | 2011 | Duodenorenal fistula | *Pediatric Radiology* | Pediatric population |
| Mohammadi | 2010 | Liver abscess due to foreign body. [Polish] | *Polski Przeglad Chirurgiczny* | Wrong indication |
| Moore | 1987 | Foreign body of the kidney due to migration of the toothpick from the intestine to the kidney. [Russian] | *Urologii* | Pediatric population |
| Newman | 2004 | Toothpick foreign body perforation and migration mimicking Crohn's disease in a child | *Journal of Pediatric Gastroenterology and Nutrition* | Pediatric population |
| Nowatorski | 2003 | FOREIGN-BODY PERFORATION OF THE ILEUM | *British Journal of Clinical Practice* | Wrong indication |
| Nusratulloev | 2007 | Toothpick impaction with localized sigmoid perforation: Successful colonoscopic management | *Digestive Endoscopy* | Duplicate study data |
| O'Gorman | 1996 | Perforation of terminal ileum by a toothpick | *British journal of hospital medicine* | Pediatric population |
| Ohri | 1990 | Esophageal Perforation Successfully Treated With EVT | *ACG Case Rep. J.* | Wrong patient population |
| Oku | 2010 | Duodenal perforation due to an ingested lollipop stick in a 7-year-old boy | *Journal of Pediatric Surgery Case Reports* | Pediatric population |
| Ozen | 2020 | Recurrent Unexplained Cystitis Due to an Ingested Foreign Body-An Extremely Rare Case | *Urology* | Wrong indication |
| Panneerselvam | 2022 | Endocarditis presenting as back pain | *Emergency Medicine* | Wrong indication |
| Park | 2019 | Toothpick ingestion causing duodenal perforation | *Pediatric Emergency Care* | Pediatric population |
| Parry | 2018 | Ammi visnaga: Tooth-pick Ammi. [French] | *Phytotherapie* | Wrong study design |
| Perzanowski | 2006 | Cocktail stick injuries: delayed diagnosis of a retained foreign body | *British Medical Journal* | Wrong patient population |
| Ragazzi | 2010 | Ileal perforation due to an ingested fragment of a skewer: Preoperative ultrasonographic diagnosis | *Journal of Ultrasound in Medicine* | Pediatric population |
| Rahal | 2021 | Foreign body in the Eustachian tube: case presentation and technique used for removal | *Rev Bras Otorrinolaringol (Engl Ed)* | Wrong patient population |
| Rand | 1987 | Surgically treated perforations of the gastrointestinal tract caused by ingested foreign bodies | *Colorectal Disease* | Wrong study design |
| Rathaus | 2006 | S3062 Floss Your Pylorus: Accidental Ingestion of Dental Floss Pick With Pyloric Injury | *Official journal of the* | Wrong patient population |
| RibeiroFde | 2008 | Endoscopic management of colonic perforation due to wooden toothpick | *Surgical Endoscopy and Other Interventional Techniques* | Duplicate study data |
| Rodriguez-Hermosa | 2008 | More on ingestion of a toothpick | *New England Journal of Medicine* | Wrong study design |
| Saini | 2020 | More on ingestion of a toothpick | *N Engl J Med* | Wrong study design |
| Saini | 2020 | Floss Your Pylorus: Accidental Ingestion of Dental Floss Pick With Pyloric Injury | *American Journal of Gastroenterology* | Wrong patient population |
| Sarici | 2017 | Time for remicade? An unusual cause of recurrent abdominal pain | *American Journal of Gastroenterology* | Duplicate study data |
| Schoffstall | 1989 | An infant case of hypopharyngeal foreign body of a food pick | *Practica Oto-Rhino-Laryngologica* | Pediatric population |
| Schoffstall | 1989 | Case report of a wooden toothpick found in the renal pelvis at operation | *Journal of Urology* | Wrong indication |
| Sealock | 2012 | Minimally Invasive Management of Gastrointestinal Foreign Bodies | *Pediatric Endosurgery and Innovative Techniques* | Pediatric population |
| Sekine | 2019 | Accidentally ingested toothpicks causing severe gastrointestinal injury: a practical guideline for diagnosis and therapy based on 136 case reports | *World Journal of Surgery* | No original data |
| Senger | 1933 | Clinical analysis of 124 cases of upper gastrointestinal tract foreign bodies in a general Hospital | *Journal of Gastroenterology and Hepatology (Australia)* | Wrong study design |
| Spilde | 2004 | Delayed ileal perforation following lollipop-stick ingestion in a two year old | *Journal of Pediatric Surgery Case Reports* | Pediatric population |
| Suganuma | 2015 | Uncommon cause of chronic abdominal pain in 11 years old boy. [Czech] | *Ceska a Slovenska Gastroenterologie a Hepatologie* | Pediatric population |
| Swedan | 2022 | An interesting presentation of foreign body ingestion | *American Journal of Gastroenterology* | Duplicate study data |
| Uehara | 2010 | Management of ingested foreign objects and food bolus impactions | *Gastrointestinal Endoscopy* | Wrong study design |
| Usta | 2016 | An alcoholic with hematochezia from an accidental cocktail sword ingestion | *American Journal of Gastroenterology* | Wrong patient population |
| Volf | 2005 | Toothpick perforation of a colon diverticulum: An adjunct autopsy finding | *Medicine, Science and the Law* | Wrong study design |
| Waintraub | 2015 | Re: Duodeno-iliac fistula secondary to ingested toothpick | *ANZ Journal of Surgery* | Wrong study design |
| Weiss | 1996 | A Patient with Acute Abdominal Pain Caused by an Unnoticed Swallowed Toothpick Misdiagnosed as Acute Appendicitis | *Arch Iran Med* | Pediatric population |
| Weissberger | 1991 | Foreign bodies in the gastro-intestinal tract | *South African Journal of Surgery* | Wrong study design |
| Wilcher | 2010 | Kidney injury and hematuria due to duodenal perforation by an ingested toothpick | *Journal of Gastroenterology and Hepatology* | Duplicate study data |
| Yagnik | 2019 | Endoscopic management of foreign bodies in the upper gastrointestinal tract in South China: a retrospective study of 561 cases | *Dig Dis Sci* | Wrong study design |
| Yao | 2022 | Laminectomy for Penetrating Spinal Cord Injury with Retained Foreign Bodies | *Orthop Surg* | Wrong patient population |
| Yu | 2021 | Endoscopic removal of sharp-pointed foreign bodies with both sides embedded into the duodenal wall in adults: A retrospective cohort study | *International Journal of General Medicine* | Wrong study design |
| Zeng | 2013 | Clinical analysis of 32 children with perforation caused by foreign body in upper gastrointestinal tract | *Chinese Journal of Applied Clinical Pediatrics* | Wrong study design |
| Zhang | 2010 | Penetrating Toothpick in the Portal Vein Resulting in Refractory Multifocal Liver Abscesses | *ACG Case Reports Journal* | Wrong patient population |
| Zhang | 2022 | Unusual differential diagnosis of upper abdominal pain | *Diagnostic & Therapeutic Endoscopy* | Duplicate study data |
| Zhou | 2020 | Intrapleural Foreign Body: Case Report | *Respiration* | Wrong patient population |
| Excluded Studies from Citation Chasing | | | | |
| A | 1981 | Large foreign bodies of the gastrointestinal tract | *International surgery* | Wrong patient population |
| Alhadeff | 1955 | Perforation of Meckel's diverticulum by foreign body and review of the literature | *The British journal of surgery* | Wrong patient population |
| Arango | 2011 | Incidental foreign body in the gastrointestinal tract: Report of three cases and literature review | *Rev Col Gastroenterol* | Wrong patient population |
| As | 1912 | FOREIGN BODIES IN THE INTESTINE | *British medical journal* | Wrong patient population |
| Balogun | 2022 | An unusual cause of pyogenic liver abscess. The conundrum of broom splinter. Report of two cases | *Nigerian Journal of Medicine* | Wrong patient population |
| Barrett | 1950 | Foreign bodies in the cardiovascular system | *The British journal of surgery* | Wrong study design |
| Behzad | 2020 | Interesting coincidence of liver abscess secondary to foreign body and gastric inflammatory fibroid polyp | *The Turkish journal of gastroenterology* | Inadequate Information |
| Ben-Ishay | 2017 | Trans-colonic foreign body penetration of the retro-hepatic vena cava. Report of a case and review of the literature | *Trauma case reports* | Wrong patient population |
| Bloom | 1986 | Foreign bodies of the gastrointestinal tract | *Annals of Surgery* | Wrong study design |
| Bonomaully | 2021 | Foreign body perforation of the distal ileum: an unusual cause of right iliac fossa pain managed via single incision laparoscopic surgery (SILS) approach | *BMJ case reports* | Wrong patient population |
| Boyse | 2001 | US OF SOFT-TISSUE FOREIGN BODIES AND ASSOCIATED COMPLICATIONS WITH SURGICAL CORRELATION | *Radiographics* | No original data |
| Brofman | 2006 | Evaluation of Bowel and Mesenteric Blunt Trauma with Multidetector CT | *Radiographics* | No original data |
| Byrne | 1994 | Foreign bodies, bezoars, and caustic ingestion | *Gastrointestinal endoscopy clinics of North America* | No original data |
| Carrera | 2011 | Absceso hepatico como complicacin diferida a cuerpo extra gastrointestinal | *Gastroenterologia y hepatologia* | Wrong patient population |
| Chaikhouni | 1985 | Foreign bodies of the esophagus | *The American surgeon* | Wrong patient population |
| Chauvin | 2012 | Management and endoscopic techniques for digestive foreign body and food bolus impaction | *Digestive and liver disease* | Wrong study design |
| Chen | 2015 | A comparative study of small intestinal perforation secondary to foreign body and other non-traumatic causes | *Ulusal travma ve acil cerrahi dergisi* | Wrong study design |
| Clarkston | 1992 | Gastrointestinal foreign bodies. When to remove them, when to watch and wait | *Postgraduate medicine* | Wrong study design |
| Cortas | 2011 | Pancreatitis aguda de etiologa atpica | *Revista clinica espanola* | Wrong patient population |
| Coughlin | 1977 | Obstruction of the gastrointestinal tract by foreign bodies in adults | *The Medical journal of Australia* | Wrong patient population |
| 仰亮 王 | 2021 | A Case of Intrahepatic Foreign Body Treated by Laparoscopy Combined with C-Type Arm Liver Partial Resection | *Advances in Clinical Medicine* | Inadequate Information |
| Deniz | 2022 | CT Evaluation of Swallowed Foreign Bodies Located in the Gastrointestinal System | *Cureus* | Wrong study design |
| Drnovsek | 1999 | Gastrointestinal case of the day. Chronic ileocolocolic intussusception secondary to a mobile cecum and a benign fibrovascular mass | *Radiographics* | No original data |
| E | 1975 | Ingested foreign bodies | *New York state journal of medicine* | Wrong patient population |
| Erbil | 2013 | Emergency admissions due to swallowed foreign bodies in adults | *World journal of gastroenterology* | Wrong study design |
| Ergul | 2008 | A transverse colonic mass secondary to Actinomyces infection mimicking cancer | *The Turkish journal of gastroenterology Gastroenterology* | Wrong patient population |
| Evans | 2015 | Intentional ingestions of foreign objects among prisoners: A review | *World journal of gastrointestinal endoscopy* | No original data |
| Fa | 1991 | Foreign body in the intestinal tract with penetration into the liver | *Der Chirurg; Zeitschrift fur alle Gebiete der operativen Medizen* | Wrong patient population |
| Fasuliak | 1995 | A foreign body as the cause of the occurrence of liver abscesses | *Klinichna khirurhiia* | Wrong patient population |
| Fell | 1948 | Foreign bodies | *The Surgical clinics of North America* | No original data |
| Fm | 1976 | Intestinal perforation in adults due to ingested opaque foreign bodies | *The American journal of gastroenterology* | Wrong patient population |
| Fujino | 1988 | ENDOSCOPIC REMOVAL OF A DUODENAL FOREIGNBODY : REPORT OF TWO CASES | *Acta Gastro-enterologica Belgica* | Wrong patient population |
| G | 1990 | An exceptional case of foreign body-induced pancreatitis | *The American journal of gastroenterology* | Wrong patient population |
| Garc­a | 1980 | Foreign bodies in the gastrointestinal tract | *Revista espanola de las enfermedades del aparato digestivo* | Wrong patient population |
| Garc­a | 2013 | Extraccian endoscapica de cuerpo extra enclavado en sigma | *Gastroenterologia y hepatologia* | Wrong study design |
| Garc­a | 2015 | Absceso hepatico atapico: cuerpo extra hepatico | *Revista clinica espanola* | Inadequate Information |
| Garg | 2013 | Duodeno-hepatic penetration by a swallowed traditional wooden toothbrush: a case report | *OA Case Reports* | Wrong patient population |
| Gayer | 2011 | Foreign objects encountered in the abdominal cavity at CT | *Radiographics* | No original data |
| Georgeades | 2021 | Primary Aortoduodenal Fistula: A Case Report and Current Literature Review | *Annals of vascular surgery* | Wrong patient population |
| Gharbi | 1994 | A rare cause of liver abscess | *La Tunisie medicale* | Wrong patient population |
| Gibert | 2000 | Cuerpos extraos esofagogastricos: factors de riesgo, manifestaciones clanicasy actiyudes teraputicas | *Medicine - Programa de Formacian Medica Continuada Acreditado* | Wrong patient population |
| Gilbert | 1990 | The role of ultrasound in the detection of non-radiopaque foreign bodies | *Clinical radiology* | Wrong study design |
| Grokhovskiĭ | 1987 | Rare case of a foreign body of the liver | *Klinicheskaia khirurgiia* | Wrong patient population |
| Hb | 1967 | Foreign body perforation of Meckel's diverticulum | *The American surgeon* | Wrong patient population |
| Hermosa | 2001 | Perforaciones intestinales causadas por cuerpos extraos | *Ciruga Espanola* | Wrong patient population |
| Hong | 2015 | Risk factors for complications associated with upper gastrointestinal foreign bodies | *World journal of gastroenterology* | Wrong study design |
| Hu | 2022 | Evaluation of the risk factors for severe complications and surgery of intestinal foreign bodies in adults: a single-center experience with 180 cases | *Gastroenterology report* | Wrong study design |
| Hung | 2007 | Rare cause of abdominal pain in a healthy woman | *Southern medical journal* | Wrong patient population |
| Isik | 2014 | How could such a wide piece of tree root pass through the narrow pyloric orifice? An extremely rare case | *The American journal of case reports* | Wrong patient population |
| Jackson | 1957 | Foreign bodies in the esophagus | *American journal of surgery* | Wrong study design |
| Jamal | 2013 | Successful laparoscopic removal of an ingested toothbrush | *Journal of surgical technique and case report* | Wrong patient population |
| John | 1996 | Perforation of the gastro-intestinal tract by a foreign body. A case report | *South African journal of surgery. Suid-Afrikaanse tydskrif vir chirurgie* | Wrong patient population |
| Karamarkovic | 2007 | Hepatic abscess secondary to a rosemary twig migrating from the stomach into the liver | *World journal of gastroenterology* | Wrong patient population |
| Khelfa | 2012 | Removal of Penetrating Sharp-Pointed Objects from the Stomach and Duodenum | *Int J Gen Med.* | Wrong study design |
| Kim | 2007 | Journey of a swallowed toothbrush to the colon | *The Korean journal of internal medicine* | Wrong patient population |
| KIRSNER | 1949 | GASTROENTEROLOGY: A Review of the Literature from July 1947 to July 1948 | *Archives of internal medicine* | Wrong study design |
| Kl | 2012 | Foreign Body-Induced Liver Abscess: Is Surgery Indispensable? | *Journal of Clinical Case Reports* | Inadequate Information |
| Klein | 2012 | Intentional ingestion and insertion of foreign objects: a forensic perspective | *The journal of the American Academy of Psychiatry and the Law* | Wrong patient population |
| Koornstra | 2008 | Management of rectal foreign bodies: description of a new technique and clinical practice guidelines | *World journal of gastroenterology* | Wrong study design |
| Kumar | 2000 | Foreign bodies migrating from gut to liver | *Indian journal of gastroenterology* | Wrong patient population |
| Lai | 2003 | Risk factors predicting the development of complications after foreign body ingestion | *The British journal of surgery* | Wrong study design |
| Lawhorne | 1979 | Occult liver abscess and foreign body perforation of the bowel | *The American surgeon* | Wrong patient population |
| Lee | 2006 | A case of colohepatic penetration by a swallowed toothbrush | *World journal of gastroenterology* | Wrong patient population |
| Lee | 2019 | Retrospective analysis of endoscopic management of foreign bodies in the upper gastrointestinal tract of adults | *Journal of the Chinese Medical Association* | Wrong study design |
| Lerche | 1911 | THE ESOPHAGOSCOPE IN REMOVING SHARP FOREIGN BODIES FROM THE ESOPHAGUS | *Journal of the American Medical Association* | Wrong patient population |
| Lheureux | 1996 | Ingestion de corps Atrangers: attitude pratique | *Ranimation Urgences* | Wrong study design |
| Li | 2006 | Endoscopic management of foreign bodies in the upper-GI tract: experience with 1088 cases in China | *Gastrointestinal endoscopy* | Wrong study design |
| Llompart | 2002 | Abordaje endoscapico de los cuerpos extraos esofagicos. Resultados de una serie retrospectiva de 501 casos | *Gastroenterologia y hepatologia* | Wrong study design |
| Marks | 1986 | Emergency gastrointestinal endoscopy and endoscopy for the emergency department | *Emergency medicine clinics of North America* | No original data |
| Mellado | 2012 | Uncommon Causes of Acute Abdominal Pain: Multidetector Computed Tomography Pearls and Pitfalls for the Radiologist on Call | *Current problems in diagnostic radiology* | Wrong study design |
| Memon | 2002 | Accidental ingestion of cotton bud stick during alcohol intoxication: an unusual cause of caecal perforation | *Irish medical journal* | Wrong patient population |
| Merino | 2016 | Cuerpo extrano: una causa infrecuente de absceso hepatico | *Gastroenterología y Hepatología* | Wrong patient population |
| Mikkelsen | 1982 | Foreign bodies in the gastrointestinal tract. Report from prisoners in the Copenhagen prisons during the period 1965-1969 and 1975-1979 | *Ugeskrift for laeger* | Wrong patient population |
| Miranda | 2018 | Perforacion de intestino delgado por cuerpo extraniƒo:: hallazgos en el TC | *SERAM* | Wrong study design |
| MACKBY | 1948 | Foreign body in second portion of duodenum perforating pelvis of right kidney | *Journal of the Mount Sinai Hospital, New York* | Wrong patient population |
| Mosca | 2001 | Endoscopic management of foreign bodies in the upper gastrointestinal tract: report on a series of 414 adult patients | *Endoscopy* | Wrong study design |
| O’Sullivan | 1996 | Deliberate ingestion of foreign bodies by institutionalised psychiatric hospital patients and prison inmates | *Irish journal of medical science* | Wrong study design |
| O'Brien | 1954 | PERFORATION OF THE INTESTINE BY INGESTED FOREIGN BODIES | *Medical Journal of Australia* | Wrong patient population |
| O'Flynn | 2007 | Fish bones and other foreign bodies | *Clinical otolaryngology and allied sciences* | Wrong study design |
| Omoya | 2013 | Twig tea impaction in the colon causing abdominal pain | *Clinical journal of gastroenterology* | Wrong patient population |
| Ooi | 1998 | MANAGEMENT OF ANORECTAL FOREIGN BODIES: A CAUSE OF OBSCURE ANAL PAIN | *ANZ Journal of Surgery* | Wrong study design |
| Palta | 2008 | Foreign-body ingestion: characteristics and outcomes in a lower socioeconomic population with predominantly intentional ingestion | *Gastrointestinal endoscopy* | Wrong study design |
| Pietrabissa | 1999 | Laparoscopic removal of an ingested foreign body that had migrated into the liver | *Endoscopy* | Wrong patient population |
| Pinto | 2004 | Pictorial essay: foreign body of the gastrointestinal tract in emergency radiology | *La Radiologia medica* | Wrong study design |
| Rajagopalan | 1982 | Free perforation of the small intestine | *Annals of surgery* | Wrong study design |
| Ribas | 2014 | Ingested foreign bodies: do we need a specific approach when treating inmates? | *The American surgeon* | Wrong study design |
| Ricote | 1985 | Fiberendoscopic removal of foreign bodies of the upper part of the gastrointestinal tract | *Surgery, gynecology & obstetrics* | Inadequate Information |
| Rokutan | 2006 | NADPH oxidases in the gastrointestinal tract: a potential role of Nox1 in innate immune response and carcinogenesis | *Antioxidants & redox signaling* | Wrong study design |
| Ruan | 2020 | Retrospective observational analysis of esophageal foreign bodies: a novel characterization based on shape | *Scientific reports* | Wrong study design |
| Ryan | 1987 | Septic discitis--a misnomer | *The Medical journal of Australia* | Wrong patient population |
| Sanchez-Muaoz | 2010 | Absceso hepaitico secundario a la impactaciÃ³n de un cuerpo extrao en la pared colanica | *Revista clinica espanola* | Wrong study design |
| Sarmast | 2012 | Gastrointestinal Tract Perforations Due to Ingested Foreign Bodies; A review of 21 cases | *British Journal of Medical Practitioners* | Wrong study design |
| Satoh | 1985 | FIBERENDOSCOPIC REMOVAL OF UPPER GASTROINTESTINAL FOREIGN BODIES | *Acta Gastro-enterologica Belgica* | Wrong patient population |
| Schneider | 1982 | Foreign body aspiration and ingestion during dental treatment | *The Compendium of continuing education in dentistry* | Wrong study design |
| Schwesinger | 1989 | Endoscopic diagnosis and treatment of mucosal lesions of the esophagus | *The Surgical clinics of North America* | No original data |
| Sethi | 1994 | Deep neck abscesses changing trends | *The Journal of laryngology and otology* | Wrong study design |
| Sharma | 2015 | Spontaneous Trans-Abdominal Expulsion of a Foreign Body: A Rare Occurrence | *Journal of clinical and diagnostic research : JCDR* | Wrong patient population |
| Singh | 1997 | Complications Associated with 327 Foreign Bodies of the Pharynx, Larynx, and Esophagus | *The Annals of otology, rhinology, and laryngology* | Wrong study design |
| Sockeel | 2009 | Perforations de lâ Aophage thoracique par corps Atranger | *Journal de chirurgie* | Wrong patient population |
| Soga | 2022 | Accidental duodenal foreign body of toothbrush removed laparoscopically: a case report | *Surgical case reports* | Wrong patient population |
| Soong | 1990 | Self-mutilating behaviour and deliberate ingestion of foreign bodies | *The Ulster medical journal* | Wrong study design |
| Sowa | 1979 | FIBERENDOSCOPIC EXTRACTION OF FOREIGN BODIES IN THE STOMACH ”CASE REPORT AND THE REVIEW OF THE LITERATURE” | *Acta Gastro-enterologica Belgica* | Wrong patient population |
| Suzuki | 2011 | A case of perforative peritonitis caused by a piece of bamboo in a patient on peritoneal dialysis | *Clinical and experimental nephrology* | Wrong patient population |
| Syrakos | 2008 | Surgical Intervention for Gastrointestinal Foreign Bodies in Adults: A Case Series | *Medical principles and practice : international journal of the Kuwait University, Health Science Centre* | Wrong study design |
| Tromans | 2018 | Deliberate ingestion of foreign bodies as a form of self-harm among inpatients within forensic mental health and intellectual disability services | *The Journal of Forensic Psychiatry & Psychology* | Wrong study design |
| Uomo | 1994 | Necrotizing acute pancreatitis due to a common bile duct foreign body | *The American journal of gastroenterology* | Wrong patient population |
| Vemula | 1982 | Colonoscopic removal of a foreign body causing colocutaneous fistulas | *Gastrointestinal endoscopy* | Wrong patient population |
| Vizcarrondo | 1983 | Foreign bodies of the upper gastrointestinal tract | *Gastrointestinal endoscopy* | Wrong study design |
| Votey | 1989 | Emergency ear, nose, and throat procedures | *Emergency medicine clinics of North America* | No original data |
| Wakayama | 1994 | A case of phlegmonous esophagitis associated with diffuse phlegmonous gastritis | *The American journal of gastroenterology* | Inadequate information |
| Wang | 2020 | Misdiagnosis of peripheral abscess caused by duodenal foreign body: a case report and literature review | *BMC gastroenterology* | Wrong patient population |
| Wang | 2021 | Upper gastrointestinal foreign bodies in adults: A systematic review | *The American journal of emergency medicine* | Wrong study design |
| Ward | 1978 | Migration into the Liver by Ingested Foreign Body | *International Journal of Clinical Practice* | Wrong patient population |
| Webb | 1995 | Management of foreign bodies of the upper gastrointestinal tract: update | *Gastrointestinal endoscopy* | Wrong study design |
| Webb | 1988 | Management of foreign bodies of the upper gastrointestinal tract | *Gastroenterology* | Wrong study design |
| Weiland | 2002 | Conservative management of ingested foreign bodies | *Journal of gastrointestinal surgery* | Wrong study design |
| Werth | 1990 | A safe and quick method for endoscopic retrieval of multiple gastric foreign bodies using a protective sheath | *Surgery, gynecology & obstetrics* | Wrong study design |
| Wt | 1966 | Foreign-body perforation of the intestine | *The Medical journal of Australia* | Wrong patient population |
| Wu | 2010 | Endoscopic management of suspected esophageal foreign body in adults | *Diseases of the esophagus* | Wrong study design |
| Xuereb | 2013 | Liver abscess following ingestion of a foreign object | *NA* | Wrong study design |
| Yagmur | 2009 | Distal ileal perforation secondary to ingested foreign bodies | *Journal of the College of Physicians and Surgeons--Pakistan : JCPSP* | Wrong patient population |
| Yao | 2015 | Endoscopic Management of Foreign Bodies in the Upper Gastrointestinal Tract of Adults | *BioMed research international* | Wrong study design |
| Yuan | 2017 | Endoscopic management of foreign bodies in the upper gastrointestinal tract: An analysis of 846 cases in China | *Experimental and therapeutic medicine* | Wrong study design |
| Zamary | 2017 | This too shall pass: A study of ingested sharp foreign bodies | *The journal of trauma and acute care surgery* | Wrong study design |
| Zhu | 2016 | Successful Endoscopic Management of Pancreatic Foreign Body | *The American journal of the medical sciences* | Duplicate study data |
| Zimmermann | 2016 | Tumor-Like Lesions of the Hepatobiliary Tract Caused by Gallstones, Foreign Bodies, and Bile | *Springer Reference Live* | Wrong study design |
| Zissin | 2008 | Abdominal CT findings in small bowel perforation | *The British journal of radiology* | Wrong study design |

**Supplementary Table 8.** Country of publication.

| Country/area | Number of cases | % |
| --- | --- | --- |
| Algeria | 1 | 0.2 |
| Argentina | 2 | 0.4 |
| Australia | 14 | 2.9 |
| Austria | 5 | 1.0 |
| Belgium | 4 | 0.8 |
| Brazil | 5 | 1.0 |
| Bulgaria | 2 | 0.4 |
| Cameroon | 1 | 0.2 |
| Canada | 15 | 3.1 |
| Chile | 5 | 1.0 |
| China | 40 | 8.4 |
| Colombia | 3 | 0.6 |
| Congo | 1 | 0.2 |
| Costa Rica | 1 | 0.2 |
| Croatia | 3 | 0.6 |
| Czech Republic | 1 | 0.2 |
| Denmark | 5 | 1.0 |
| Ecuador | 2 | 0.4 |
| France | 5 | 1.0 |
| Germany | 22 | 4.6 |
| Ghana | 1 | 0.2 |
| Greece | 8 | 1.7 |
| Hong Kong | 4 | 0.8 |
| India | 8 | 1.7 |
| Iran | 1 | 0.2 |
| Ireland | 1 | 0.2 |
| Israel | 1 | 0.2 |
| Italy | 29 | 6.1 |
| Japan | 35 | 7.3 |
| Jordan | 1 | 0.2 |
| Korea | 19 | 4.0 |
| Lebanon | 1 | 0.2 |
| Luxembourg | 1 | 0.2 |
| Malaysia | 1 | 0.2 |
| Mexico | 1 | 0.2 |
| Netherlands | 2 | 0.4 |
| New Zealand | 1 | 0.2 |
| Nigeria | 1 | 0.2 |
| Pakistan | 1 | 0.2 |
| Poland | 2 | 0.4 |
| Portugal | 2 | 0.4 |
| Romania | 6 | 1.3 |
| Saudi Arabia | 2 | 0.4 |
| Serbia | 1 | 0.2 |
| Spain | 11 | 2.3 |
| Switzerland | 8 | 1.7 |
| Syria | 1 | 0.2 |
| Taiwan | 23 | 4.8 |
| Tunisia | 1 | 0.2 |
| Turkey | 9 | 1.9 |
| United Arab Emirates | 1 | 0.2 |
| United Kingdom | 13 | 2.7 |
| United States of America | 143 | 29.9 |
| Uruguay | 1 | 0.2 |
| Vietnam | 4 | 0.8 |

**Supplementary Table 9.** Publication year and format.

| Item | Number of cases | % |
| --- | --- | --- |
| Year of case publication |  |  |
| 1910–1919 | 1 | 0.2 |
| 1920–1929 | 1 | 0.2 |
| 1930–1939 | 3 | 0.6 |
| 1940–1949 | 10 | 2.1 |
| 1950–1959 | 8 | 1.7 |
| 1960–1969 | 10 | 2.1 |
| 1970–1979 | 21 | 4.4 |
| 1980–1989 | 37 | 7.7 |
| 1990–1999 | 43 | 9.0 |
| 2000–2009 | 95 | 19.8 |
| 2010–2019 | 167 | 34.9 |
| 2020–present | 83 | 17.3 |
| Publication format |  |  |
| Full text study or case report | 393 | 82.0 |
| Letter to the editor | 30 | 6.3 |
| Image | 28 | 5.8 |
| Abstract | 32 | 6.7 |

**Supplementary Table 10.** Additional locations of the ingested foreign bodies.

| Location | Number of cases | % |
| --- | --- | --- |
| Lung^a^ | 4 | 0.8 |
| Bladder | 4 | 0.8 |
| Left portal vein | 4 | 0.8 |
| Ischiorectal fossa | 3 | 0.6 |
| Aorta | 2 | 0.4 |
| Scrotum | 2 | 0.4 |
| Renal vein^b^ | 2 | 0.4 |
| Right ureter | 2 | 0.4 |
| Right paracolic gutter | 2 | 0.4 |
| Perianal region | 2 | 0.4 |
| Piriform sinus^b^ | 2 | 0.4 |
| Left diaphragm | 2 | 0.4 |
| Meckel diverticulum | 2 | 0.4 |
| Right sacral nerves | 1 | 0.2 |
| Left common iliac vein | 1 | 0.2 |
| Pelvic cavity | 1 | 0.2 |
| Left pelvic sidewall | 1 | 0.2 |
| Anti-mesenteric border of the terminal ileum | 1 | 0.2 |
| Parapharyngeal space | 1 | 0.2 |
| Pharyngo-esophageal junction | 1 | 0.2 |
| Left renal artery | 1 | 0.2 |
| Splenic artery | 1 | 0.2 |
| Spleen | 1 | 0.2 |
| Left ovary | 1 | 0.2 |
| Gastrocolic ligament | 1 | 0.2 |
| Mesenteric lymph node | 1 | 0.2 |
| Right upper quadrant space | 1 | 0.2 |
| Right iliac fossa | 1 | 0.2 |
| Right parasternal space | 1 | 0.2 |
| Sternothyroid muscle | 1 | 0.2 |
| Perineum | 1 | 0.2 |
| Buttock | 1 | 0.2 |
| Nose | 1 | 0.2 |
| Abdominal mass | 1 | 0.2 |
| Right sacrospinalis muscle | 1 | 0.2 |

^a^Three of four cases involving the right side.

^b^Cases involved both left and right side.

**Supplementary Table 11.** Patient demographics and details of sharp wooden object ingestions stratified by development of adverse events.

| Variable | No adverse events | | Adverse events | | *P* value |
| --- | --- | --- | --- | --- | --- |
|  | Number of cases^a^ | Results (%) | Number of cases^a^ | Results (%) |  |
| Age, years | 31 |  | 442 |  | 0.10 |
| Mean |  | 48.2 |  | 53.3 |  |
| Median (IQR) |  | 50 (38–58) |  | 55 (42–64) |  |
| Range |  | 20–76 |  | 18–93 |  |
| Sex | 29 |  | 446 |  | 0.03 |
| Male |  | 15 (51.7) |  | 318 (71.3) |  |
| Race/ethnicity | 2 |  | 47 |  |  |
| White/Caucasian |  | 2 (100) |  | 34 (72.3) |  |
| Black |  | 0 |  | 8 (17.0) |  |
| Asian |  | 0 |  | 4 (8.5) |  |
| Hispanic |  | 0 |  | 1 (2.1) |  |
| Comorbidities | 31 |  | 448 |  | 0.42 |
| Substance use disorder |  | 2 (6.5) |  | 44 (9.8) |  |
| Edentulous |  | 1 (3.2) |  | 28 (6.3) |  |
| Depression |  | 1 (3.2) |  | 7 (1.6) |  |
| Psychosis |  | 0 |  | 5 (1.1) |  |
| Intellectual disability |  | 0 |  | 4 (0.9) |  |
| Dementia |  | 0 |  | 4 (0.9) |  |
| Pica |  | 0 |  | 2 (0.4) |  |
| Anxiety |  | 0 |  | 2 (0.4) |  |
| Sharp wooden object ingested | 31 |  | 448 |  | <0.01 |
| Toothpick |  | 21 (67.7) |  | 422 (94.2) |  |
| Skewer |  | 2 (6.5) |  | 9 (2.0) |  |
| Chopstick |  | 5 (16.1) |  | 5 (1.1) |  |
| Cocktail stick |  | 0 |  | 8 (1.8) |  |
| Wood stick needle |  | 3 (9.7) |  | 0 |  |
| Lollipop stick |  | 0 |  | 1 (0.2) |  |
| Wooden bowel fragment |  | 0 |  | 1 (0.2) |  |
| Matchstick |  | 0 |  | 1 (0.2) |  |
| Pointy wood shard |  | 0 |  | 1 (0.2) |  |
| Knowledge of ingestion | 25 |  | 300 |  | 0.02 |
| Aware at time of presentation |  | 9 (36.0) |  | 35 (11.7) |  |
| Recalled ingestion after diagnosis |  | 1 (4.0) |  | 37 (12.3) |  |
| Unaware |  | 15 (60.0) |  | 228 (76.0) |  |

IQR, interquartile range.

^a^Reflects the number of cases for which the data was reported.

**Supplementary Table 12.** Endoscopic tools used for removal of foreign body.

| Endoscopic tools^a^ | Number of cases | % |
| --- | --- | --- |
| Snare | 42 | 28.0 |
| Forceps | 86 | 57.3 |
| Overtube | 3 | 2.0 |
| Clips | 22 | 14.7 |
| Over-the-scope clips | 3 | 2.0 |
| Less commonly used tools |  |  |
| Insulation-tipped knife | 2 | 1.3 |
| Potassium titanyl phosphate laser | 1 | 0.7 |
| Dormia basket | 1 | 0.7 |
| Basket catheter | 1 | 0.7 |
| Flex knife | 1 | 0.7 |
| Hook knife | 1 | 0.7 |
| Biliary balloon dilator and stents | 1 | 0.7 |
| Grasper | 1 | 0.7 |
| Foreign body clamp | 1 | 0.7 |
| Latex hood | 1 | 0.7 |
| Transparent cap-fitting device | 1 | 0.7 |

^a^Percentages are out of the 150 cases in which the foreign body was removed; 30 cases did not describe how the foreign body was removed, but six cases reported clip placement afterwards

**Supplementary Figure 1.** Assessment of methodological quality of included studies.
