# Supplementary figures and images for: Clinical features and endoscopic management of sharp wooden object ingestions: a systematic review of 479 cases
Source: Gastroenterol Rep (Oxf). 2025 May 14;13:goaf035. doi: 10.1093/gastro/goaf035 (PMC12073997; doi:10.1093/gastro/goaf035)

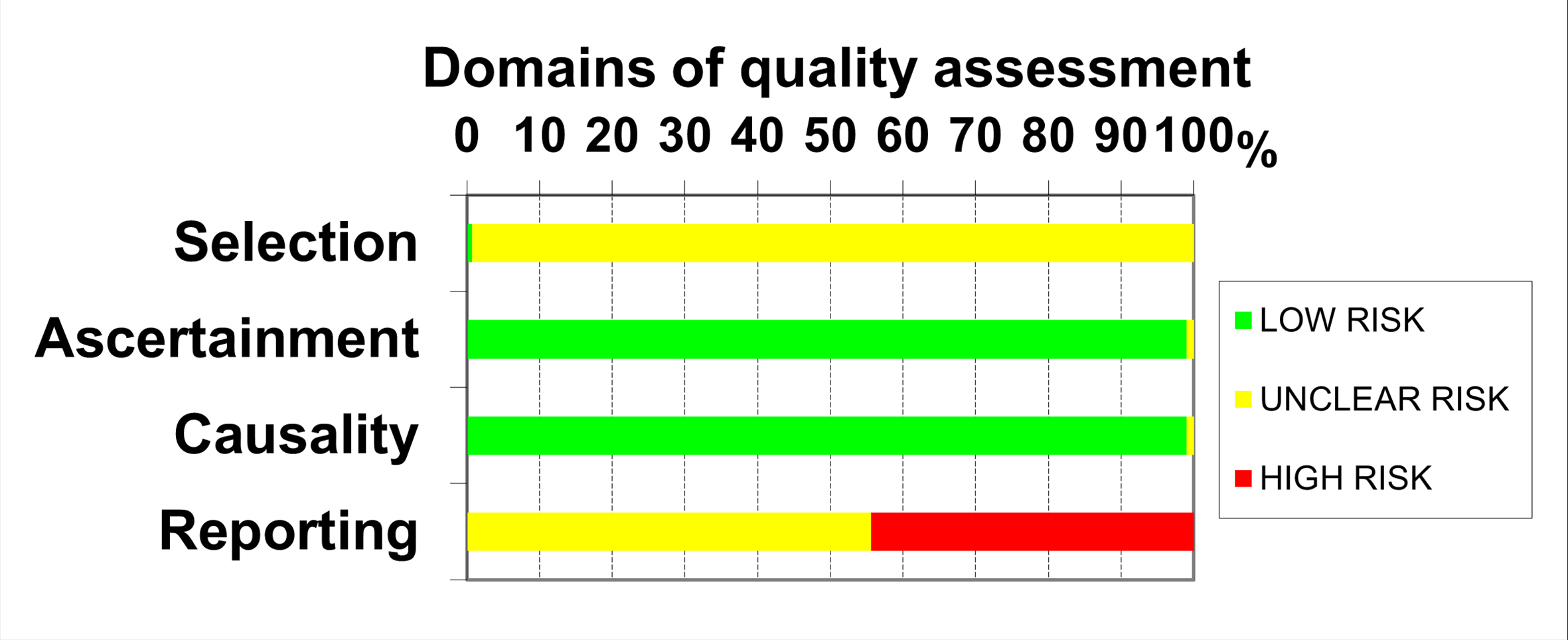

Supplement: goaf035_Supplementary_Data [file goaf035_supplementary_data.zip › Supplemental_Figure_1.tif]
